# Bayesian modeling suggests that IL-12 (p40), IL-13 and MCP-1 drive murine cytokine networks *in vivo*

**DOI:** 10.1186/s12918-015-0226-3

**Published:** 2015-11-09

**Authors:** Sarah L. Field, Tathagata Dasgupta, Michele Cummings, Richard S. Savage, Julius Adebayo, Hema McSara, Jeremy Gunawardena, Nicolas M. Orsi

**Affiliations:** Women’s Health Research Group, Section of Cancer & Pathology, Leeds Institute of Cancer & Pathology, St James’s University Hospital, Leeds, LS9 7TF UK; Department of Systems Biology, Harvard Medical School, 200 Longwood Avenue, Boston, Massachusetts 02115 USA; Systems Biology Centre, University of Warwick, Coventry, CV4 7AL UK; IDeA Labs, Department of Computer Science, TMCB 1124, Brigham Young University, Provo, UT 84602 USA

**Keywords:** Bayesian network, Cytokine, Hormone, Lactation, Mouse

## Abstract

**Background:**

Cytokine-hormone network deregulations underpin pathologies ranging from autoimmune disorders to cancer, but our understanding of these networks in physiological/pathophysiological states remains patchy. We employed Bayesian networks to analyze cytokine-hormone interactions *in vivo* using murine lactation as a dynamic, physiological model system.

**Results:**

Circulatory levels of estrogen, progesterone, prolactin and twenty-three cytokines were profiled in post partum mice with/without pups. The resultant networks were very robust and assembled about structural hubs, with evidence that interleukin (IL)-12 (p40), IL-13 and monocyte chemoattractant protein (MCP)-1 were the primary drivers of network behavior. Network structural conservation across physiological scenarios coupled with the successful empirical validation of our approach suggested that *in silico* network perturbations can predict *in vivo* qualitative responses. *In silico* perturbation of network components also captured biological features of cytokine interactions (antagonism, synergy, redundancy).

**Conclusion:**

These findings highlight the potential of network-based approaches in identifying novel cytokine pharmacological targets and in predicting the effects of their exogenous manipulation in inflammatory/immune disorders.

**Electronic supplementary material:**

The online version of this article (doi:10.1186/s12918-015-0226-3) contains supplementary material, which is available to authorized users.

## Background

Cytokines comprise an extensive array of extracellular protein mediators best known for their traditional immunoregulatory functions, although their multiple roles in the orchestration of an array of physiological and pathological processes such as cancer, autoimmunity and cardiovascular disease are now well-recognized [1–10]. Many former reductionist studies have attempted to ascribe given physiological effects to the actions of one or small numbers of cytokines, but this strategy has met with conceptual difficulties stemming from the fact that their actions can be paradoxical at different concentrations and differ according to the prevailing hormonal milieu [3, 11]. Furthermore, the mechanistic insight offered by such studies in terms of relative cytokine interactions is also limited given that physiological responses are seldom governed by any one cytokine but rather by the combined influences of many. In this respect, these mediators are believed to operate as part of highly complex networks, wherein they exhibit antagonism, synergy and functional redundancy [12–14]. It is therefore clear that gaining a fuller understanding of cytokine function in any biological or clinical scenario rests with clarifying their interactions at a network level rather than relying on the increasingly inadequate T helper cell type 1/2 (Th1/Th2) paradigm [15].

**Fig. 1:**
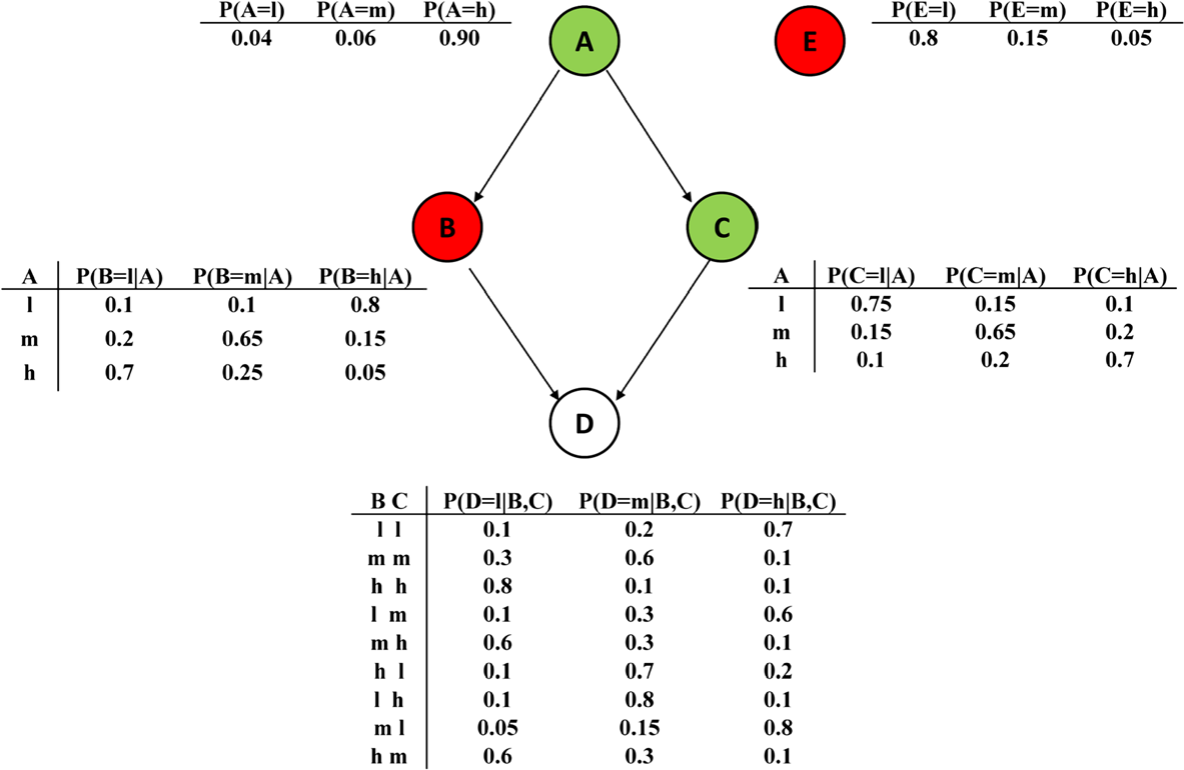
Illustrative Bayesian network describing causal relationships between five variables, with their associated conditional probability tables. The values of each variable have been discretized into low (l), medium (m) and high (h) bins. The notation P(B = l|A) refers to the probability of B being in the low value bin, conditional upon the value of A (which itself can be l, m or h). Note that with one parent only (i.e. the case for nodes B and C), both row and column probabilities sum to 1, whereas with multiple inputs (i.e. in the case of D), only the rows sum to 1. The nodes are colored green (high), white (medium) and red (low) to illustrate *in silico* perturbation where A falls into a high concentration bin (probability 0.9) and E falls into a low concentration bin (probability 0.8). The implications of this are demonstrated through the conditional probability tables associated with each downstream node wherein having D in a given concentration state is dictated by the particular combination of states of its parents B and C as dictated by their corresponding overall (marginal) probabilities (the entries as captured in the conditional probability tables do not change on intervention), as summarized in the histogram attached to each node. Note that the overall marginal probability of D being in a particular state (which changes under intervention) is the sum as summarized in the histogram attached to each node. Readers more familiar with Bayesian networks will note that the sub-network structures (B → A, A → C, B → D, C → D) and (C → A, A → B, B → D, C → D) are both Markov equivalent with the present one (A → B, A → C, B → D, C → D). When we condition on A, the other nodes B and C become independent no matter whether A is a tail-to-head or tail-to-tail intermediate node (this scenario differs from that seen with head-to-head node D). In other words, these three sub-networks specify the same independent assumptions belonging to the same equivalence class and the true causal network can possibly be any one of the sub-network solutions. However, causal networks based on observation alone (i.e. without intervention, which is an important tool for inferring causality) can be still partially constructed. In the present study, prior knowledge seed network edge directionalities were assigned by a modified depth-first search algorithm which helped to choose the sub-network from an equivalence class containing more than one Markov equivalent member as suggested here (see later)

Recent developments in high-throughput analytical platforms have revolutionized the quality and quantity of data available from *in vivo* experiments. The large number of analytes measurable in single samples provides the opportunity to explore their interrelationships in both physiological and pathophysiological processes [16, 17]. In this regard, Bayesian networks provide an attractive methodology for analyzing such complex biological data [18–20]. Given that many biologists are unlikely to be familiar with probabilistic graphical models, a word of introduction to Bayesian networks is warranted. A Bayesian network is a directed acyclic graph whose nodes are the variables of interest (herein, a cytokine/hormone), each of which can have a range of quantitative values which are typically discretized into a small number of bins, such as ‘low’, ‘medium’ and ‘high’. The directed edges in the graph (represented as arrows between nodes) reflect likely causal relationships between nodes. The nature of these causal relationships is captured by the graph’s underlying conditional probability table (CPT) which details the probabilities for any given node to fall into each of the different (in this case, concentration) bins given the status of its parent nodes (i.e. those directly upstream). The underlying CPT does not change upon perturbation; rather the marginal probability of that node displaying a certain behavior changes (Fig. 1). The illustrative Bayesian network shown in Fig. 1 has five variables (vertices/“nodes”). Node E is not causally influenced by any of the others, nor does it causally influence them, so this node has no edges entering or leaving it (i.e. it is ‘orphaned’). By contrast, nodes B and C are solely influenced by A, so each has a single connecting edge. However, they respond to A in quite different ways. Based on the conditional probability tables, if the value of A is categorized into a low concentration bin, then B has a marginal probability of 0.8 of falling into a high concentration bin, while C has a probability of 0.75 of falling into a low one. The status of D is influenced by both B and C and accordingly has two incoming edges. This approach offers the scope for an intricate description of D’s behavior, based on the conditional probabilities associated with the allocation of its data to high or low concentration bins which, in turn, depends on the state of A, B and C. The conditional probability tables for each node represent relative (rather than absolute) concentrations.

A Bayesian network can be inferred from experimental data through the correlations between experimentally-measured quantitative values of different nodes. Various machine learning techniques are used to undertake this inference process, which is often helped by the use of a prior-knowledge graph ‘seed’ incorporating well-recognized, literature-derived information which reduces the computational outlay required to learn networks from biological data. Such prior knowledge speeds up the search and avoids local minima, improving performance and yielding statistically more robust networks, as described in Djebbari and Quackenbush (2008) [21]. Moreover, this bias does not limit the process to learn new interactions between the nodes. Accordingly, Bayesian networks are well adapted to noisy data, small sample sizes and, most importantly, a lack of detailed knowledge about how causal interactions are implemented at a biological level. Moreover, they also allow the effect of network perturbations to be explored *in silico* [18, 19, 22, 23]. Indeed, once the network structure is established, the impact of *in silico* perturbation of upstream nodes (i.e. by changing the values in the conditional probability bins for one or more nodes) can be tracked through the network structure to assess the changes in the conditional probabilities of the downstream nodes, thereby leading to specific, experimentally verifiable predictions. It is nonetheless possible to conceive more complex biological relationships which cannot be distilled into a Bayesian acyclic graph. In this respect, Bayesian networks are acyclic insofar as they do not allow for the portrayal of structural causal loops such as those which may arise from feedback relationships [24]. However, they offer a sensible compromise between capturing complex causal relationships and computational feasibility, and have thus been used in a wide variety of scientific contexts [25–28].

While most Bayesian-based studies have focused on core biological processes using data from cell lines or tissue-based genome-wide expression microarrays, less attention has been paid to physiological or clinically relevant *in vivo* systemically-derived data [21, 29]. As such, Bayesian methodologies offer a valuable opportunity to define the nature of both known and novel mediator interrelationships in complex physiological processes such as immunity and inflammation. They are, however, correlative and their significance rests on the ability to draw causal inferences about the underlying biology from these relationships.

In order to validate the models learned through this methodology, both from a biological as much as from an analytical perspective, we developed two independent circulatory cytokine and hormone-based datasets: one drawn from lactating mice and the other from mice whose pups were removed at birth. With regard to the former network, murine lactation represents a unique model system to explore the dynamic physiological interactions between the hormonal environment and inflammation/immune function, with particular regard to the effects of prolactin (PRL), both a key driver of lactation and putative critical immunomodulator [30–33]. Since lactation PRL levels can conveniently be abrogated *in vivo* by removal of the suckling stimulus, this offers an excellent strategy to verify predictions made by *in silico* perturbation of the lactation network by comparing it with an independently-generated *in vivo* pup-free network with a physiologically-induced PRL abolition. These networks were further used to infer functionally significant nodes, such as ‘hubs’ and ‘drivers’, as well as characterizing the relationships between nodes, such as synergy and antagonism. To the best of our knowledge, there have been no previous attempts to employ Bayesian network analysis to unravel dynamic protein interactions based on physiological *in vivo* data in such a mechanistic and qualitatively validated manner (i.e. including *in vivo* confirmation of *in silico* perturbation-dependent predictions).

## Results

**Fig. 2:**
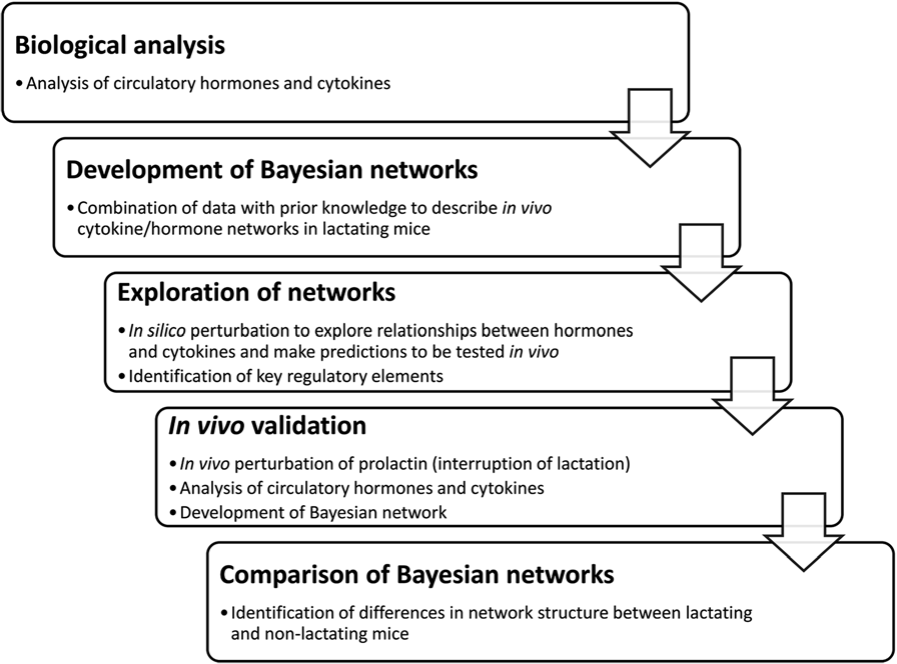
Experimental methodology utilized in the present study; initial biological analysis (via fluid-phase multiplex immunoassay, ELISA and radioimmunoassay) was followed by development and exploration of Bayesian networks. *In vivo* physiological perturbation was performed to validate the *in silico* networks.

We chose to characterize the physiological putative changes in, and interactions between, systemic hormone and cytokine concentrations using murine lactation as a model, since this system offered the benefit of featuring both intimate and highly variable physiological immunoendocrine interactions [34–39]. The benefits of this approach included not having to use multiple pharmacological agents/antibodies whose effects would require titrating to concentration (likely in a non-linear manner), and not being subject to non-physiological interactions or inducing global changes unrelated to the system of interest. Similarly, our approach deliberately aimed to avoid using multiple knockout models on both pragmatic and functional grounds given the compensatory redundancy of cytokine networks and/or the unknown postpartum traits of such models. Our design was based on two independent data sets: one covering cytokine and hormonal profiles over 7 time points throughout lactation in order to generate a Bayesian model of mediator interactions, and another abrogated lactation data set which provided a biological validation platform for assessing the predictive power of the former network in determining the physiological profile changes elicited by pup removal. The methodological workflow is described in Fig. 2; please see the methods section for experimental details.

### Biological analysis

#### Most circulatory cytokines and hormones vary markedly throughout lactation

Significant changes (*P* < 0.05, following correction for multiple comparisons) in concentration during lactation were noted for IL-1α, IL-2, IL-3, IL-5, IL-9, IL-10, IL-12 (p40), IL-12 (p70), IL-17, IFN-γ, G-CSF, GM-CSF, KC, MCP-1, MIP-1α, MIP-1β, RANTES, P_4_ and PRL (Table 1 and Additional file 1). Trends for IL-13 were also noted (*P =* 0.075). Cytokine levels showed a broad tendency to be increased on day 1 of lactation relative to naturally cycling (NC) concentrations, and significant increases were observed in IL-6, IL-9, IL-12 (p70), IL-13, G-CSF, GM-CSF, MCP-1, MIP-1α, MIP-1β and TNF-α. Furthermore, there was a marked trend for PRL, E_2_, IL-1α, IL-2, IL-9, IL-10, IL-12 (p40), IL-12 (p70), IL-13, eotaxin, G-CSF, IFN-γ, KC, MCP-1, MIP-1α and RANTES to decrease on day 2 of lactation. Most cytokine levels peaked at day 10 of lactation, particularly IL-2 and MCP-1, although the timing of this phenomenon differed for IL-1α (days 16–24), IL-9 (days 4–16), IL-12 (p40) (day 24), KC (day 21) and RANTES (day 16). There followed a significant decrease (IL-2, IFN-γ, G-CSF, GM-CSF, MCP-1, MIP-1α, MIP-1β; *P <* 0.05) or trend towards decreasing profiles (IL-1β, IL-3, IL-4, IL-5, IL-6, IL-10, IL-12 (p40), IL-12 (p70), IL-13, IL-17, eotaxin, KC, TNF-α) on day 16. P_4_ concentrations rose in line with those of PRL, peaked on day 4, fell to very low levels by day 16, and increased towards weaning. Weaning concentrations for E_2_ and P_4_ were similar to those of NC animals.

**Table 1:** Cytokine and hormone concentrations throughout lactation

Mediator	NC (*n* = 7)	Day 1 (*n* = 8)	Day 2 (*n* = 8)	Day 4 (*n* = 8)	Day 10 (*n* = 7)	Day 16 (n = 8)	Day 21 (n = 7)	Day 24 (n = 7)
IL-1α	0.52 ± 0.41^a^	3.40 ± 1.35^ab^	2.20 ± 0.75^ab^	7.46 ± 2.78^bc^	12.32 ± 2.42^c^	15.48 ± 2.16^c^	15.06 ± 2.85^c^	13.16 ± 1.81^c^
IL-1β	34.83 ± 11.89	50.12 ± 14.37	55.17 ± 6.45	57.21 ± 10.81	74.76 ± 6.21	51.07 ± 6.09	83.24 ± 25.51	41.59 ± 8.66
IL-2	5.67 ± 1.41^a^	12.50 ± 2.43^abc^	10.87 ± 1.31^ab^	25.25 ± 3.68^d^	36.73 ± 6.09^e^	17.75 ± 2.77^bcd^	25.38 ± 4.81^d^	18.08 ± 3.49^cd^
IL-3	1.50 ± 0.04^a^	1.90 ± 0.21^ab^	2.17 ± 0.51^ac^	2.77 ± 0.96^ad^	6.48 ± 3.33^def^	3.75 ± 0.84^bdef^	3.95 ± 1.13^af^	5.77 ± 2.36^bcef^
IL-4	0.10 ± 0.03	0.21 ± 0.06	0.19 ± 0.03	0.24 ± 0.12	0.58 ± 0.13	0.22 ± 0.05	0.53 ± 0.25	0.36 ± 0.15
IL-5	3.98 ± 0.69^a^	8.48 ± 1.50^ab^	10.32 ± 1.01^bc^	16.79 ± 2.54^d^	21.43 ± 2.24^d^	16.13 ± 2.48^cd^	17.84 ± 2.98^d^	13.79 ± 2.12^cd^
IL-6	17.08 ± 7.19^a^	119.81 ± 25.30^b^	151.52 ± 56.55^b^	164.98 ± 37.82^b^	265.95 ± 40.50^b^	151.35 ± 20.92^b^	201.91 ± 51.93^b^	154.86 ± 41.57^b^
IL-9	159.93 ± 24.69^a^	278.68 ± 28.05^bc^	236.61 ± 18.92^acd^	396.55 ± 46.52^be^	387.07 ± 28.31^ef^	394.43 ± 38.41^be^	296.10 ± 37.38^bde^	180.05 ± 20.59^a^
IL-10	5.69 ± 2.78^a^	18.95 ± 6.92^abc^	15.56 ± 6.16^ac^	27.99 ± 5.74^ad^	109.05 ± 29.77^d^	31.03 ± 4.86^cd^	45.68 ± 12.24^cd^	105.96 ± 62.76^bd^
IL-12 (p40)	164.44 ± 16.75^ab^	137.59 ± 37.23^abc^	114.44 ± 8.51^b^	247.74 ± 31.16^ac^	213.84 ± 20.94^ac^	160.99 ± 27.04^abc^	232.08 ± 11.32^cd^	259.51 ± 28.02^cd^
IL-12 (p70)	42.34 ± 17.66^a^	316.15 ± 101.70^bc^	249.58 ± 46.91^bd^	682.30 ± 173.51^be^	1222.66 ± 326.6^ef^	786.09 ± 90.51^ef^	699.93 ± 84.99^ce^	912.65 ± 283.08^cdf^
IL-13	117.81 ± 28.60^a^	324.07 ± 40.21^b^	261.46 ± 33.82^ab^	416.84 ± 67.09^b^	650.45 ± 108.10^ab^	334.77 ± 54.77^ab^	447.35 ± 86.24^b^	307.99 ± 69.89^b^
IL-17	42.17 ± 11.33^a^	155.83 ± 54.39^abc^	243.46 ± 27.76^bd^	391.00 ± 62.99^de^	477.14 ± 51.54^e^	446.35 ± 26.19^e^	441.08 ± 66.12^ce^	424.34 ± 55.23^e^
Eotaxin	115.26 ± 54.35	462.25 ± 158.06	388.22 ± 111.37	349.13 ± 55.09	517.32 ± 104.37	470.79 ± 42.03	446.18 ± 50.47	444.59 ± 33.41
G-CSF	1.28 ± 0.68^a^	25.06 ± 12.59^bcd^	11.39 ± 4.24^ac^	13.80 ± 2.29^bc^	40.38 ± 4.51^d^	20.45 ± 3.50^bc^	24.44 ± 5.15^bcd^	11.30 ± 2.23^bcd^
GM-CSF	6.62 ± 2.31^a^	22.41 ± 2.29^bc^	20.15 ± 1.84^b^	30.83 ± 5.85^cd^	44.02 ± 4.48^e^	32.71 ± 2.76^cd^	37.76 ± 4.15^de^	27.38 ± 4.46^cd^
IFN-γ	14.19 ± 3.16^a^	76.07 ± 14.31^ab^	51.22 ± 8.18^a^	150.66 ± 30.78^cd^	219.15 ± 44.86^d^	136.96 ± 14.43^bc^	160.27 ± 26.80^cd^	120.45 ± 35.03^bc^
KC	9.95 ± 1.75^a^	19.05 ± 3.24^ab^	10.36 ± 1.51^a^	22.66 ± 2.22^b^	26.26 ± 3.91^b^	22.89 ± 1.65^b^	28.51 ± 3.82^b^	20.39 ± 3.47^ab^
MCP-1	109.21 ± 11.86^a^	277.14 ± 23.85^bc^	202.25 ± 18.47^ab^	311.17 ± 36.45^c^	440.78 ± 49.29^d^	295.60 ± 33.14^c^	327.16 ± 38.26^c^	259.00 ± 40.22^bc^
MIP-1α	307.86 ± 44.49^a^	583.64 ± 48.62^b^	500.58 ± 38.00^b^	641.07 ± 66.25^bc^	813.92 ± 90.49^c^	501.03 ± 68.81^b^	672.14 ± 75.99^bc^	522.25 ± 72.06^b^
MIP-1β	17.64 ± 8.90^a^	97.42 ± 19.81^bc^	85.99 ± 17.25^c^	152.07 ± 30.82^cd^	248.47 ± 22.27^e^	165.79 ± 11.07^d^	175.62 ± 25.75^dbe^	173.97 ± 28.40^dbe^
RANTES	0.00 ± 0.00^abc^	2.21 ± 1.14^ac^	1.16 ± 0.96^a^	5.20 ± 2.82^abc^	4.79 ± 2.47^abc^	15.41 ± 3.52^b^	10.35 ± 2.82^abc^	10.73 ± 4.03^bc^
TNF-α	79.49 ± 41.61^a^	558.78 ± 154.41^b^	560.13 ± 188.57^b^	642.66 ± 169.61^b^	985.75 ± 148.06^b^	630.06 ± 43.70^b^	630.40 ± 125.34^b^	717.02 ± 173.20^b^
E_2_	230.63 ± 40.27	274.75 ± 111.88	96.75 ± 15.47	159.81 ± 28.44	150.16 ± 27.68	123.11 ± 15.37	205.83 ± 23.55	129.20 ± 26.77
P_4_	206.17 ± 46.07^ab^	106.89 ± 17.78^a^	241.39 ± 59.96^ab^	364.94 ± 58.13^b^	158.10 ± 52.16^a^	33.51 ± 5.28^c^	130.08 ± 47.18^ac^	243.94 ± 98.57^ab^
PRL	8.71 ± 4.72^ab^	78.88 ± 46.34^acd^	50.75 ± 19.27^de^	298.00 ± 79.05^f^	85.43 ± 17.26^dfg^	77.38 ± 43.77^acdeg^	1.14 ± 0.74^b^	7.00 ± 2.76^bce^

Cytokine, E_2_ (pg/ml), PRL and P_4_ (ng/ml) concentrations throughout lactation (mean ± SEM). Groups that do not share a common superscript letter are significantly different from each other (*P <* 0.05); NC - naturally cycling

#### Cytokine and hormone profiles during lactation cluster into three distinct time-series

**Fig. 3:**
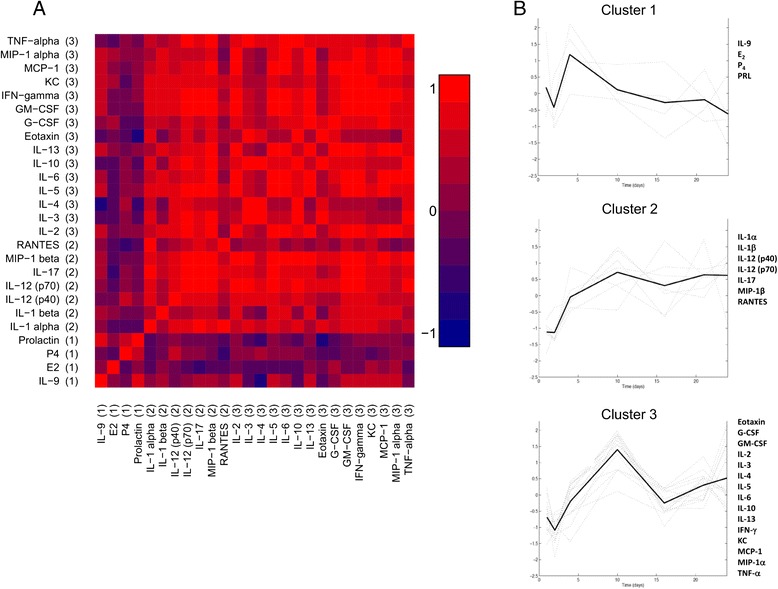
Correlations between cytokine and hormone mediators; Panel **a** Heat map demonstrating the correlations between cytokines, E_2_, P_4_ and PRL. Red coloring indicates positive correlations, while blue coloring indicates negative correlations. The strength of color reflects the strength of correlation (as indicated by the scale) – numbers in brackets refer to cluster identities. Panel **b** clusters indicating relationships between mediators. Three clusters were identified: Cluster 1 - IL-9, E_2_, P_4_, PRL; Cluster 2 - IL-1α, IL-1β, IL-12 (p40), IL-12 (p70), IL-17, MIP-1β, RANTES; and cluster 3 - eotaxin, G-CSF, GM-CSF, IL-2, IL-3, IL-4, IL-5, IL-6, IL-10, IL-13, IFN-γ, KC, MCP-1, MIP-1α, TNF-α

In order to reveal the temporal structure in the data, we examined correlations between analytes and clustered the time series. Significant correlations were noted across the array of mediators investigated, except for E_2_, IL-1β, IL-9, IL-12 (p40), P_4_ and PRL (Fig. 3, panel a). These relationships were used to inform the cluster analysis (Fig. 3, panel b) which revealed that analyte profiles fell into three clusters: Cluster 1 (IL-9, E_2_, P_4_, PRL) peaked around day 5, and tailed off steadily thereafter. Cluster 2 (IL-1α, IL-1β, IL-12 (p40), IL-12 (p70), IL-17, MIP-1β, RANTES) started off low and increased steadily to plateau from day 10 onwards, while Cluster 3 (eotaxin, G-CSF, GM-CSF, IL-2, IL-3, IL-4, IL-5, IL-6, IL-10, IL-13, IFN-γ, KC, MCP-1, MIP-1α, TNF-α) behaved similarly but had a broad peak centered on day 10 of lactation.

#### Development of Bayesian networks

##### Generation of the lactation prior network

In the search for a network graph, an initial prior network structure seed was used as a bias, and the final network was learned from the data. The space of possible networks was explored using the TabuSearch algorithm and, at each iteration, the score was evaluated by adding, removing or reversing individual edges. The introduction of a prior network seed initiated the learning stage from all 53 data samples for 26 nodes as soft bias wherein the original seed edges were judged by the score in the same way as any other network edge. For each node, all prior probabilities (as defined by the BDe scoring metric with Dirichlet distribution) exceeded zero, enabling the construction of Bayesian networks reflecting all possible nodal interactions. Initiating the parameters (conditional probabilities) for each node at the beginning was achieved using a uniform distribution; and these changed with the data. Details of the prior network (Additional file 2) revealed that this featured 21 nodes, 9 of which appeared as parents.

##### The lactation Bayesian network structure features six structural hubs

**Fig. 4:**
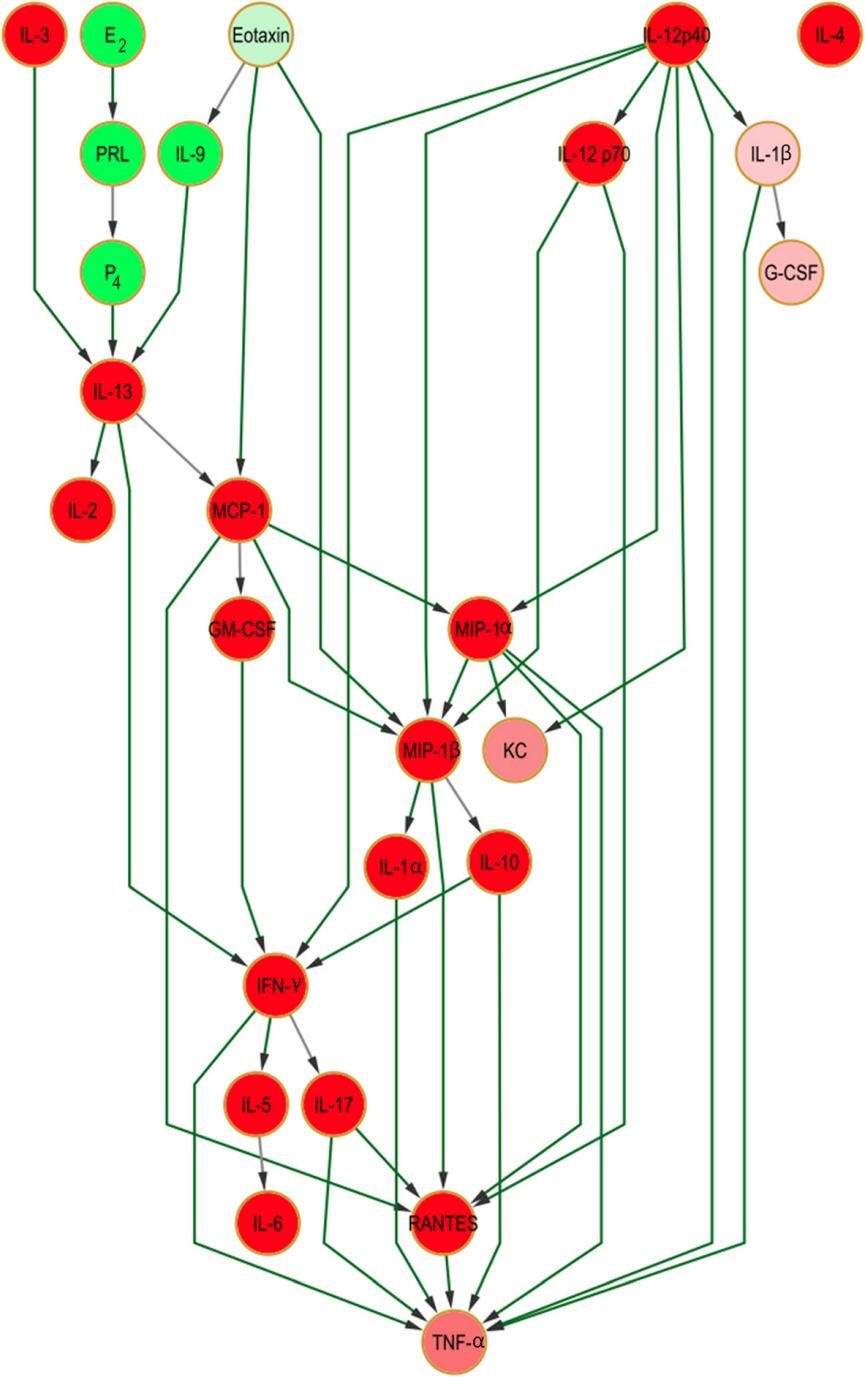
Bayesian network showing cytokine interrelationships in murine lactation. The nodes are color-coded according to the conditional probability of corresponding mediator relative concentrations being high (green), low (red) or medium (white) concentration given the state(s) of their parent nodes; however, within the present network no nodes fell into the medium category. Relative to the white color, the normalized concentration (low or high) determines the intensity of the node color. Very high confidence level edges (causal connecting lines between nodes) are colored in green (or grey if lower than the 0.9 cutoff), based upon the confidence analysis of the Bayesian result

A Bayesian network from the lactation dataset was generated incorporating a prior knowledge network (Additional file 2) with experimental data, wherein node (i.e. mediator) interactions were portrayed as directed edges implying likely causal relationships. Each node was associated with a set of conditional probabilities which determined its status (i.e. probable relative concentration) dependent upon the status of its parents. This network organized into two main branches, one with IL-3, E_2_ and eotaxin as the first-line parents and the other with IL-12 (p40) as the principal parent node (Fig. 4). The network itself was assembled around six structural hubs (i.e. possible signal integrators; defined as nodes with >1 input and output edges totaling ≥5) comprising IFN-γ, IL-13, MCP-1, MIP-1α, MIP-1β, and RANTES. The terminal node was TNF-α, which was connected - both directly and indirectly - to each of these hubs. A total of 42 directed edges (35 of which were of high confidence) representing cytokine causal relationships connected all but one node: only IL-4 was orphaned. E_2_, P_4_, and PRL had a high probability of being present at elevated concentration relative to all other network components, with the exception of eotaxin and IL-9 (Fig. 4).

There were more samples (53) than nodes (26, initially) in the present study. This compares well with previous biological studies. Djebbari and Quackenbush (2008) [21] learned a Bayesian network with 63 features from 38 samples, while Gao and Wang (2011) [40] developed a Bayesian network learned with 36 features from 46 samples. Ideally, the use of many more samples would have allowed us to create the most robust model, but this need had to be offset against making an ethical and appropriate use of animals. Nonetheless, the highly stringent bootstrapping approach used herein ensured robustness of the model. Its purpose was to resample the data with replacement in order to avoid over-fitting so as to address the issue of using a limited number of samples. The final network contained 25 nodes wherein the maximum theoretical number of parents for each node could be 24. Networks were learned for each generated pseudo-dataset. Model averaging of these was then performed to obtain edge confidence scores. The overall bootstrap confidence was estimated by evaluating how many times relative to the total number of iterations a particular feature of interest (i.e. directed edge, Markov relation, etc.) appeared. The final network had an overall confidence of 0.9 (i.e. the learned network features were present in at least 90 networks out of 100 iterations). Increasing the confidence threshold from 0.7 to 0.9 had no effect, confirming the result with high confidence (i.e. strongly supported by the data). This was further confirmed by the fact that a Variational Bayesian State Space Model (VBSSM) network (which did not account for prior knowledge of known mediator interactions in their construction) revealed conservation of the core network structure, including hubs (except MIP-1α) akin to those obtained from the seeded Bayesian models (Additional file 3). IL-10, IL-12 (p70) (a child of the seeded network parent IL-12 (p40)), IL-13, eotaxin and MIP-1β were parents, and RANTES and TNF-α were terminal nodes (with IL-1α and IL-2 as peripheral termini). These topological changes were expected given that the VBSSM network only featured a subset of the original network nodes following automated construction (Additional file 3). The inference process was performed without prior knowledge bias of mediator interactions. As indicated in Table 2, the F-score value, high number of true negatives, and the relatively high sensitivity value when compared to the seeded network in Fig. 4 show that there is a relatively high topographical similarity between the two. This is further confirmed by the retention of major regulatory hubs and the fact that all the edges associated with TNF-α are incoming.

**Table 2:** Sensitivity, specificity and F-score values for comparisons between lactation, pup-free and VBBSM networks

	VBSSM vs Seeded lactation network	VBSSM vs Seeded pup-free network	VBSSM Lactation vs VBBSM Pup-free network
Specificity	0.94	0.95	0.88
Sensitivity	0.30	0.14	0.17
F-Score	0.46	0.25	0.28
True positives	13	6	5
False positives	18	14	15
False negatives	30	36	25
True negatives	263	268	108

Bayesian networks model conditional independence so that accurately removing arcs from all possible connections is an important measurement for accuracy. This is reflected in the specificity, which is close to one if true negatives (TNs) are high and false positives (FPs) are low. For the purpose of internal validation (in addition to the experimental aspects), the VBSSM-based results obtained without prior knowledge were compared to those from seeded Bayesian learning. It is important to note that VBSSM results were derived under a strict confidence level check. However, we cannot expect a high agreement in true positives (TPs) for the structural comparisons performed for both the lactation and pup-free data but the high TN and specificity values are encouraging (first two data columns). The last column represents the network structural comparison within the VBSSM analysis between lactation and pup-free data

#### Exploration of networks

##### Perturbation of PRL in silico

PRL perturbation *in silico* in the lactation model was chosen as a starting point given that this hormone is widely considered as having potent immunomodulatory properties. Perturbation was achieved by allocating its low concentration bin a conditional probability of 1. This perturbation revealed that, apart from a fall in P_4_ profile, the downstream nodes, hubs and edges remained qualitatively unchanged (i.e. no/minimal directional shift (up or down) in conditional probabilities such that nodal status also remained unchanged (Table 3), an observation supported by the lack of correlation between PRL and the majority of other mediators (Fig. 3). The terms ‘increased’ and ‘decreased’ are used as less cumbersome terminology throughout this manuscript to describe changes in node conditional probabilities following perturbation (e.g. ‘decreased’ effectively relates to a shift in node conditional probability towards a low concentration bin). The only exceptions were IL-13, MCP-1 and IL-2, whose concentrations ‘increased’, indicating a degree of negative regulation of these cytokines by PRL (Table 3 and Additional file 4). Due to the relatively minor influence of *in silico* PRL depletion on the overall network (except, most notably, its negative regulation of the immediate downstream hubs IL-13 and MCP-1), further perturbations were performed in order to explore the relative importance of other network elements.

**Table 3:** Changes in conditional probability associated with perturbation of PRL and structural hubs

Cytokine	Low	Medium	High
PRL	0.065	0.692	0.243
P_4_	0.066	0.786	0.148
P_4-PRL_	0.778	0.111	0.111
P_4-PRL+IL-13_	0.778	0.111	0.111
P_4-PRL+MCP-1_	0.778	0.111	0.111
P_4-PRL+IL-13+MCP-1_	0.778	0.111	0.111
IL-13	0.400	0.499	0.101
IL-13_-PRL_	0.379	0.348	0.272
IL-13_-PRL+IL-13_	0.000	0.000	1.000
IL-13_-PRL+MCP-1_	0.379	0.348	0.272
IL-13_-PRL+IL-13+MCP-1_	0.000	0.000	1.000
IL-2	0.274	0.602	0.124
IL-2_-PRL_	0.274	0.481	0.246
IL-2_-PRL+IL-13_	0.111	0.111	0.778
IL-2_-PRL+MCP-1_	0.274	0.481	0.246
IL-2_-PRL+IL-13+MCP-1_	0.111	0.111	0.778
MCP-1	0.431	0.458	0.111
MCP-1_-PRL_	0.418	0.386	0.196
MCP-1_-PRL+IL-13_	0.200	0.229	0.571
MCP-1_-PRL+MCP-1_	0.000	0.000	1.000
MCP-1_-PRL+IL-13+MCP-1_	0.000	0.000	1.000
GM-CSF	0.359	0.526	0.115
GM-CSF_-PRL_	0.353	0.469	0.178
GM-CSF_-PRL+IL-13_	0.225	0.316	0.458
GM-CSF_-PRL+MCP-1_	0.111	0.111	0.778
GM-CSF_-PRL+IL-13+MCP-1_	0.111	0.111	0.778
MIP-1α	0.235	0.687	0.078
MIP-1α_-PRL_	0.250	0.644	0.106
MIP-1α_-PRL+IL-13_	0.250	0.521	0.229
MIP-1α_-PRL+MCP-1_	0.270	0.360	0.370
MIP-1α_-PRL+IL-13+MCP-1_	0.270	0.360	0.370
MIP-1β	0.607	0.208	0.186
MIP-1β_-PRL_	0.570	0.224	0.205
MIP-1β_-PRL+IL-13_	0.434	0.293	0.273
MIP-1β_-PRL+MCP-1_	0.321	0.357	0.321
MIP-1β_-PRL+IL-13+MCP-1_	0.321	0.357	0.321
KC	0.165	0.706	0.129
KC_-PRL_	0.168	0.692	0.140
KC_-PRL+IL-13_	0.183	0.635	0.181
KC_-PRL+MCP-1_	0.201	0.569	0.230
KC_-PRL+IL-13+MCP-1_	0.201	0.569	0.230
IL-1α	0.626	0.223	0.151
IL-1α_-PRL_	0.605	0.230	0.165
IL-1α_-PRL+IL-13_	0.528	0.256	0.216
IL-1α_-PRL+MCP-1_	0.465	0.281	0.254
IL-1α_-PRL+IL-13+MCP-1_	0.465	0.281	0.254
IL-10	0.648	0.230	0.122
IL-10_-PRL_	0.622	0.245	0.133
IL-10_-PRL+IL-13_	0.526	0.301	0.173
IL-10_-PRL+MCP-1_	0.448	0.348	0.204
IL-10_-PRL+IL-13+MCP-1_	0.448	0.348	0.204
IFN-γ	0.504	0.299	0.197
IFN-γ_-PRL_	0.465	0.299	0.236
IFN-γ_-PRL+IL-13_	0.325	0.337	0.338
IFN-γ_-PRL+MCP-1_	0.352	0.325	0.323
IFN-γ_-PRL+IL-13+MCP-1_	0.316	0.341	0.343
IL-5	0.517	0.327	0.156
IL-5_-PRL_	0.487	0.336	0.177
IL-5_-PRL+IL-13_	0.377	0.386	0.237
IL-5_-PRL+MCP-1_	0.398	0.375	0.228
IL-5_-PRL+IL-13+MCP-1_	0.370	0.390	0.240
IL-17	0.513	0.391	0.096
IL-17_-PRL_	0.486	0.406	0.108
IL-17_-PRL+IL-13_	0.390	0.467	0.144
IL-17_-PRL+MCP-1_	0.407	0.454	0.138
IL-17_-PRL+IL-13+MCP-1_	0.384	0.470	0.146
IL-6	0.608	0.306	0.086
IL-6_-PRL_	0.585	0.319	0.096
IL-6_-PRL+IL-13_	0.506	0.371	0.123
IL-6_-PRL+MCP-1_	0.521	0.361	0.119
IL-6_-PRL+IL-13+MCP-1_	0.501	0.374	0.124
RANTES	0.483	0.276	0.241
RANTES_-PRL_	0.454	0.287	0.259
RANTES_-PRL+IL-13_	0.373	0.322	0.305
RANTES_-PRL+MCP-1_	0.328	0.347	0.326
RANTES_-PRL+IL-13+MCP-1_	0.327	0.347	0.326
TNF-α	0.372	0.317	0.311
TNF-α_-PRL_	0.364	0.321	0.316
TNF-α_-PRL+IL-13_	0.346	0.328	0.325
TNF-α_-PRL+MCP-1_	0.341	0.331	0.329
TNF-α_-PRL+IL-13+MCP-1_	0.340	0.331	0.329

Conditional probabilities are given to each of three bins (low, medium and high). Highest conditional probability values in any given bin indicate a greater likelihood of the relevant mediator’s concentration being in that concentration bin. The large font denotes the node of interest; small font indicates the perturbed parent node; a ‘-’ sign indicates perturbation by reducing concentration while a ‘+’ sign indicates perturbation by increasing concentration

##### IL-13, MCP-1 and IL-12 (p40) are key driver nodes

As outlined above, IL-13 and MCP-1 were selected for perturbation by allocating them to a high concentration bin with a probability of 1 (based on their ~3 and 2-fold increases in high concentration conditional probabilities, respectively, following *in silico* PRL depletion) (Table 3). They were perturbed both individually and in combination. Increasing IL-13 concentration caused extensive network changes, including a shift in MCP-1 and GM-CSF to a higher concentration and IFN-γ to a moderately high concentration (Additional file 5 panel A). The effects extended as far downstream as the terminal node: KC became medium and MIP-1α, RANTES and TNF-α became more medium. Note that the term ‘medium’ is used herein as shorthand to refer to the intermediate, mid-range relative concentration ‘equal frequency’ bin based on the data discretization (i.e. the bin containing the third of samples falling in the middle of the range analyzed). Increasing MCP-1 resulted in similarly extensive changes: among its children, GM-CSF concentration increased markedly while MIP-1β and RANTES became medium (Additional file 5 panel B). Further downstream effects included MIP-1α concentration becoming high, KC moderately medium-high, and IFN-γ and TNF-α both more medium. PRL branch perturbation (combined PRL/IL-13 and PRL/MCP-1) also resulted in significant changes to downstream node conditional probabilities as far as the terminal node (Additional file 5 panel C). Similarly marked effects were noted for combined PRL/IL-13/MCP-1 perturbation, which resulted in marked changes in all downstream hub statuses and conditional probability values (Additional file 6).

As a major parent of the second network branch (i.e. the one without IL-3, E_2_ and eotaxin as first-line parents), IL-12 (p40) was chosen for perturbation. IL-12 (p40) perturbation *in silico* (by allocation to a high concentration bin) had a dramatic downstream impact: IL-1β, IL-12 (p70) and KC concentration increased, MIP-1α became moderately high, and MIP-1β, IFN-γ and TNF-α became medium (Additional file 7 panel A). Combined perturbations of both network branches (IL-12 (p40) and eotaxin) also affected common downstream nodes (Additional file 8, panels A and B).

##### MCP-1 can act synergistically with IL-13 and/or IL-12 (p40)

**Fig. 5:**
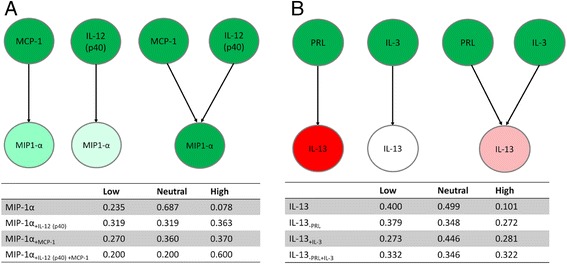
Synergistic and antagonistic relationships between mediators. Schematic representation of **a** MCP-1 and IL-12 (p40) synergy on MIP-1α with associated conditional probability table, and **b** antagonism between PRL and IL-3 on IL-13

The combined perturbation of IL-13 and MCP-1 on a background of low PRL had particularly marked effects on IFN-γ, resulting in an increase in its high concentration bin conditional probability (0.343), an effect greater than that achieved by each parent perturbation in isolation (0.338 and 0.323 from 0.236 for IL-13 and MCP-1, respectively). This suggests the presence of a synergistic interaction in which IL-13 may co-opt MCP-1 given that it is also one of its parents (Table 3). However, more striking still were the synergistic effects of MCP-1 and IL-12 (p40) in relation to MIP-1α (Fig. 5). Conditional probabilities in the MIP-1α high concentration bin were much greater when both were combined (0.363 and 0.370 compared to 0.600 in combination). Comparable, though less striking effects were noted for MIP-1β when IL-12 (p40) and MCP-1 were perturbed together, and for IFN-γ when IL-12 (p40) and IL-13 were perturbed in combination (Additional file 9).

##### IL-3 and PRL are potentially antagonistic

Similar perturbations of the parent node IL-3 were also performed. Allocation of IL-3 to low, medium and high concentration bins affected IL-2, IL-13 and MCP-1 concentrations to some degree, although the most striking feature of these changes was that the greatest effects were noted when IL-3 concentration was medium, rather than high or low (i.e. its effects were not linearly related to concentration; Additional file 10). Perturbing IL-3 by allocating it to a high concentration bin resulted in an increase in IL-13 concentration, as did perturbing PRL to a low concentration. This suggested that, as parents, IL-3 and PRL may be antagonistic in terms of their effects on IL-13, a notion supported by the intermediate conditional probabilities for IL-13 concentration resulting from high levels of both these parents (Fig. 5).

##### MIP-1β exhibits a biphasic response to eotaxin

Allocating eotaxin to a high concentration bin had minor effects, causing little more than a shift in MIP-1β towards a higher concentration (Additional file 9 and Additional file 11 panel A). By contrast, allocating eotaxin to a low concentration bin had marked effects on its children: IL-9 concentration was reduced whereas MCP-1 and MIP-1β became more medium, an effect carried through downstream to GM-CSF (Additional file 11 panel B). Intriguingly, the shift in MIP-1β concentration was in the same direction, independent of whether eotaxin concentration was perturbed upwards or downwards, suggesting a concentration-dependent biphasic response.

#### In vivo validation

##### Cytokine profiles fall when lactation is not established

**Fig. 6:**
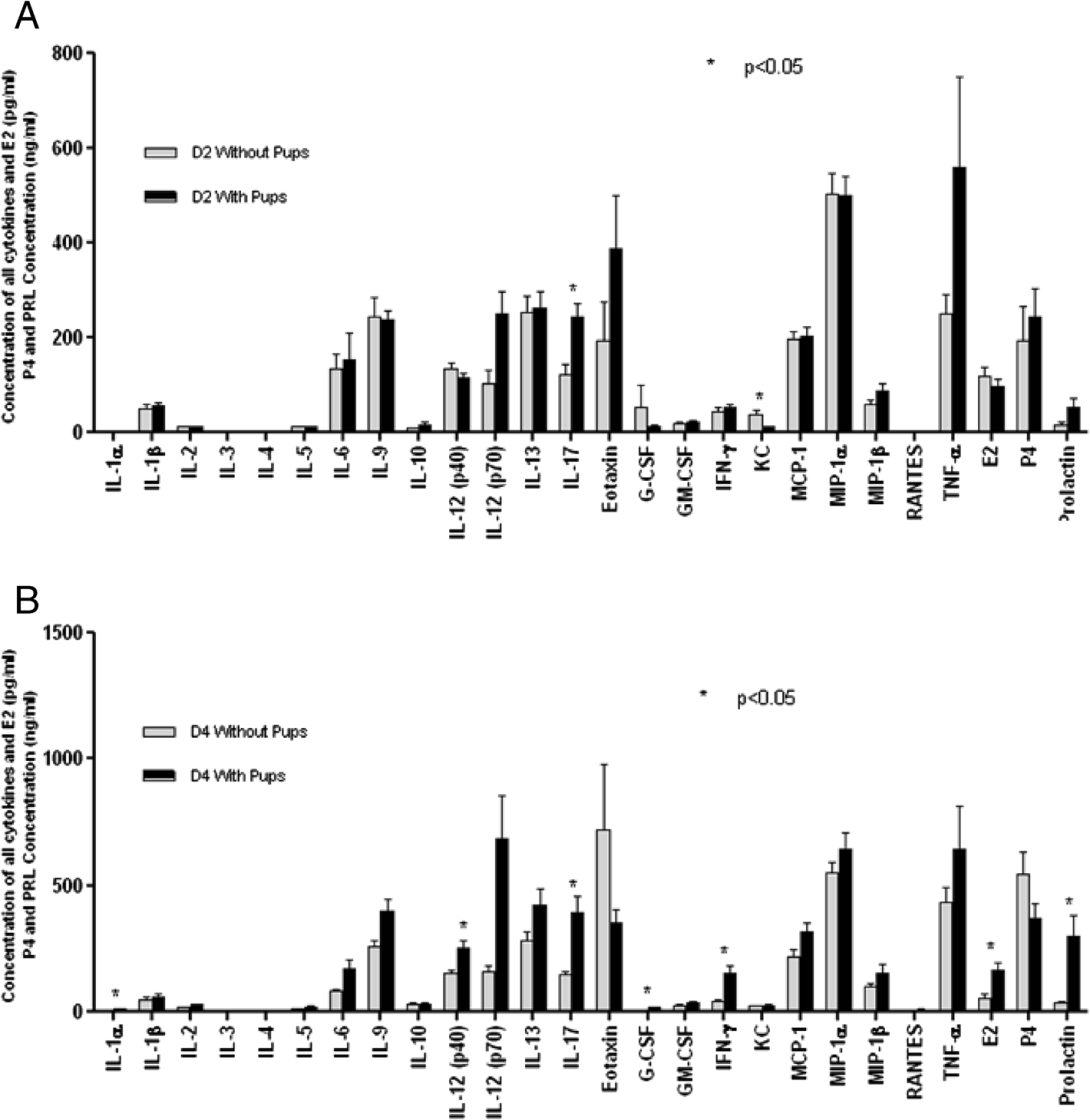
Systemic cytokine profiles following parturition with and without lactation. Comparison of cytokine and PRL/P_4_/E_2_ concentrations in mice with/without pups on **a** day 2 and **b** day 4 of lactation (*P < 0.05)

In order to determine experimentally the impact of lactation on cytokine and hormone profiles relative to a baseline, samples were obtained from dams whose pups were removed at birth (i.e. from animals which would not have exhibited a lactation-dependent rise in PRL). This physiological perturbation resulted in a fall in maternal serum concentrations of IL-17 and a rise in KC on day 2 (corrected *P <* 0.05) (Fig. 6). By day 4 post-partum, the differences between females with and without pups were more pronounced: IL-1α, IL-12 (p40), IL-17, IFN-γ, G-CSF, E_2_ and PRL levels were significantly higher in nursing dams (*P <* 0.05). Similar trends were also noted for IL-2, IL-5, IL-9 and IL-12 (p70).

#### Comparison of Bayesian networks

##### Lactation and pup-free networks share striking similarities

**Fig. 7:**
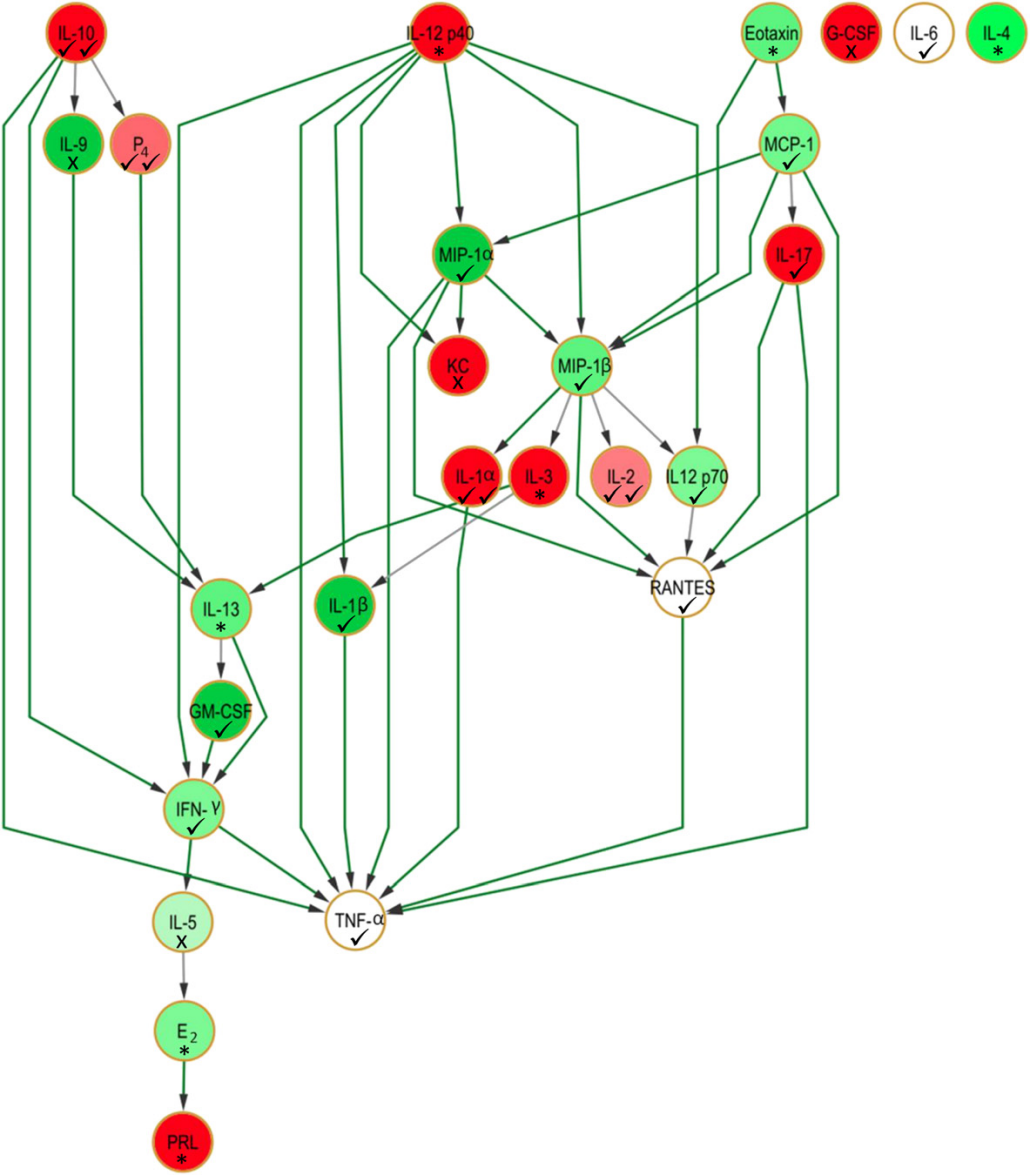
Bayesian network displaying cytokine interrelationships associated with pup-free dams *in vivo*. Color coding of both nodes and edges is as described for Fig. 4. Symbols indicate if the nodal concentration bin was correctly categorized by *in silico* perturbation. ✓✓ - Correctly/closely categorized by PRL perturbation alone. ✓ - Correctly/closely categorized by PRL perturbation in combination with IL-12 (p40), IL-13 and MCP-1, or by any other single mediator perturbation. X – Not correctly categorized by any perturbation. * - Could not be evaluated due to being upstream of the perturbations performed

Strikingly, the pup-free dam Bayesian network retained the same core structural hubs as the lactation network (IFN-γ, IL-13, MCP-1, MIP-1α, MIP-1β, and RANTES), with IL-12 (p40) as the principal parent and TNF-α as the terminal node, despite expected differences in network topology (Fig. 7). This second network featured an additional parent (IL-10), three orphan nodes (G-CSF, IL-4 and IL-6) unconnected to the main network and a total of 42 edges (32 of high confidence) which connected 23 of the 26 nodes. When the lactation and pup-free dam networks were compared using accepted measures of network comparison (see Experimental Procedures), the F-score was 0.861 and the total complexities of these networks were 379 and 375, respectively. These scores imply a striking similarity in terms of topology and complexity between both networks despite their being generated from entirely independent data sets.

As noted for the lactation network, the VBSSM variant for pup-free dams retained many of the core features of its Bayesian counterpart, with the exception of eotaxin which became a terminal node (Additional file 3, panel B). IL-12 (p40) and IL-10 were principal parents, with IFN-γ and MIP-1β displayed as structural hubs. As with the earlier case, the inference process was performed without bias of any prior knowledge of the interactions between the cytokines being studied. The associated F-core of 0.28 (as displayed in Table 2) is low, although this was expected given the relatively low number of edges and nodes observed (under very high confidence criteria) in comparison to the seeded network obtained in Fig. 4. The high true negative values and specificity (i.e. true negative rate) as well as the conservation of the major regulatory hubs again point to the relatively high topological similarity between the VBSSM and seeded networks (Table 2).

##### *In silico* perturbation can correctly predict in vivo responses

The most striking difference between the two physiological networks was that half of the nodes had moved to high concentration bins (a relative observation) in the pup-free scenario, echoing the node status distribution in the *in silico* perturbed network driven by high concentration IL-13 and MCP-1 (Fig. 7 and Additional file 5). Based on a comparison between predicted (Additional file 6) and monitored (Fig. 7) effects, PRL perturbation alone only categorized four nodes correctly/closely (P_4_, IL-1α, IL-2 and IL-10 to low concentration bins). However, combined PRL/IL-13/MCP-1 perturbation correctly classified the qualitative (i.e. quantitatively high/medium/low concentrations relative to the equal frequency bins) nodal status of P_4_, IL-1α, IL-10 (low concentration), RANTES, TNF-α (medium), IFN-γ (moderately high) and GM-CSF (high). Close categorization was noted for MIP-1β (predicted to be medium instead of moderately high), IL-6 (moderately low instead of medium), IL-17 (moderately low instead of low) and MIP-1α (moderately high instead of high) (Table 3 and Fig. 7). In addition to having MCP-1 as a parent, MIP-1α, MIP-1β and IFN-γ were also children of IL-12 (p40), and perturbing both of these parents by allocating them to a high concentration bin *in silico* led to their correct categorization in the pup-free network (Additional file 7 panel B and Fig. 7). Only the qualitative profile of G-CSF, KC, IL-5 and IL-9 in the pup-free network could not be predicted/validated by *in silico* perturbation of the lactation network (i.e. 79 % of nodal statuses were correctly predicted by the perturbations performed). Thus, the relative changes of levels (in both positive and negative directions ) as encoded in the probability tables broadly agreed with overall states of the nodes in the physiologically perturbed pup-free network model.

## Discussion

This study aimed to characterize physiological cytokine:cytokine and cytokine:hormone interactions in murine lactation as a model of inflammatory/immune mediator regulation using a Bayesian network-based approach. This method revealed a robust lactation network structure featuring two main branches organized around principal parents (IL-12 (p40), IL-3, E_2_ and eotaxin), structural hubs (IFN-γ, IL-13, MCP-1, MIP-1α, MIP-1β, and RANTES) and a terminal node (TNF-α). These pivotal roles resonate with the central role reported for these mediators in the control of cytokine networks: IFN-γ in atherogenesis [41], IL-12 (p40) in those governing T cell and macrophage responses [42], IL-13 in the pathophysiology of ulcerative colitis [43], IFN-γ, IL-12 (p40/p70) and MCP-1 in Erdheim-Chester disease [44] and eotaxin, IFN-γ, MIP-1α and MIP-1β in asthma [45]. A second network was generated from data drawn from pup-free dams in which removal of the suckling stimulus physiologically prevented the rise in PRL levels characteristic of the onset of lactation. This second Bayesian network, which validated *in vivo* the predictions made *in silico*, had broadly similar topology (F score 0.861) and total network complexity and, most significantly, featured the same conserved structural hubs. These findings suggest that these hub nodes connected by high confidence edges may act as structural lynchpins around which both networks assemble, potentially playing a role in integrating diverse upstream cytokine-mediated signals to induce coordinated downstream responses. *In silico* network perturbation allowed a more detailed analysis of the relative contribution of various nodes to the control of the overall network behavior. The results also pointed to the presence of ‘driver’ nodes (nodes which, when perturbed, propagated maximum amounts of changes in terms of the number of downstream nodes affected). In this respect, one branch parent (IL-12 (p40)) and two structural hubs (IL-13 and MCP-1) were responsible for orchestrating the most significant changes within the network, thereby reinforcing the proposed concept of a signal integration role played by the latter two cytokines.

The original premise underlying the choice of lactation as a model system was that this physiological setting would feature marked changes in PRL, a hormone that has been proposed as a central regulator of cytokine networks [30]. Surprisingly, PRL functioned neither as a parent nor as a structural hub, and its only connection to the rest of the network via P_4_ was through a weak confidence edge. However, the cluster analysis demonstrated that most cytokine profiles clustered separately and peaked later than PRL suggesting that these may be independently regulated *in vivo*, as reflected by the Bayesian analysis. Furthermore, when PRL was perturbed (reduced) *in silico*, there were, with the notable exception of P_4_, only minor qualitative shifts in conditional probability values in downstream mediators (including those for IL-13 - PRL’s connection to the rest of the lactation network - and MCP-1). These findings point to a limited role for PRL as an immunoregulator in murine lactation, the presence of extensive functional redundancy for its actions and/or, possibly, reflect the fact that its purported effects on cytokine profiles are largely drawn from the *in vitro* setting. Our findings are consistent with the observation that both PRL and PRL receptor knockout mice do not feature substantial immune dysfunction [46, 47]. The effects of PRL on MCP-1 are rather more unclear: in contrast to our findings, existing data implicate PRL as an inducer of MCP-1 in ovarian luteolysis and lactational bone resorption [48, 49], although the systemic/circulatory relationship between these mediators in response to the prolactinaemia of lactation remains unknown. Nevertheless, its effect on P_4_ supports PRL’s reported role in increasing serum P_4_ in rats [50].

*In silico* perturbation of downstream IL-13, by contrast, had marked effects on network response, suggesting that this cytokine had a threshold conditional probability at which downstream signaling was achieved which PRL alone as an IL-13 input function was unable to achieve. It is tempting to speculate that oxytocin, a lactation-related neurohypophyseal hormone which causes smooth muscle contraction during the let-down reflex, may represent an additional input given that the highest cytokine concentrations coincided with the period of maximal suckling/milk production [51–55]. This would be consistent with oxytocin’s known modulation of cytokine production and receptor-dependent activity in a range of settings [56, 57], and the ability of IL-13 to modulate oxytocin receptor expression [58].

A principal aspect of this study focused on the ability of *in silico* perturbation to predict cytokine levels in a different physiological scenario *in vivo*. This premise’s biological validation was achieved by comparing *in silico* lactation network behavior following perturbation induced by pup removal and the consequent abrogation of lactation *in vivo*. Strikingly, allocation of the driver nodes IL-13 and MCP-1 to high concentration bins *in silico* (akin to what occurred physiologically in the *in vivo* pup-free network) correctly (or closely) predicted the relative concentration status of 13 out of their 14 downstream cytokines *in vivo*. IL-13 and MCP-1 perturbation less accurately predicted MIP-1α and MIP-1β’s response to pup removal (based on their conditional probabilities). However, they were correctly categorized when their direct driver node parent (IL-12 (p40) and MCP-1) perturbations were used instead. These findings are consistent with the documented effects of MCP-1 induction of MIP-1α expression in murine aneurysm models and total IL-12 induction of MIP-1α in isolated human natural killer cells (albeit in the presence of IL-15) [59, 60]. By contrast, *in silico* perturbation failed to predict the cytokine concentration response of the peripheral terminal cytokine KC correctly. These findings may be accounted for by the possibility that despite KC being connected to its parents via high confidence edges in the lactation network, its regulation may be under the control of additional mediators and/or involve changes in cell-specific receptor expression not measured as part of this investigation.

*In silico* lactation network perturbation also pointed to a recognized feature of cytokine interactions: synergy (the joint action of two or more cytokines which, when acting in concert, potentiate each other’s effects). Previous studies have found IL-13 to be a selective inducer of MCP-1 [61] as supported by the present data. Furthermore, we noted an additional and previously unreported synergistic relationship between these mediators in relation to IFN-γ concentration. When IL-13 and MCP-1 were perturbed together (i.e. both allocated to high concentration bins), this resulted in a greater increase in IFN-γ concentration than that observed when either was perturbed alone. Further, more striking evidence of synergy was noted following the combined perturbations of both network branches. IL-12 (p40) and MCP-1 perturbation independently increased the concentration of their direct child MIP-1α, but this effect was markedly greater when these parents were perturbed together. Analogous, though less pronounced synergistic interactions were also noted for combined IL-12 (p40) and IL-13 perturbation which, independently, are both known to increase IFN-γ concentration [62, 63]. To the best of our knowledge, these synergistic functional relationships have not previously been reported in the literature and will form the basis of our future investigations into how widespread/conserved these relationships occur across different physiological/pathological settings, body compartments (particularly in relation to immune privileged sites) and species.

*In silico* lactation network perturbation also revealed another functional property of inflammatory networks: antagonism (the opposing/modulatory action of one cytokine on another). In this regard, IL-3 and PRL had opposing actions on IL-13, with IL-3 seemingly increasing IL-13 concentrations (in line with its documented effects on cultured murine bone marrow cells [64]), such that when these parents were perturbed together (low PRL and high IL-3), the effect on IL-13 was greater than those induced by the independent perturbation of its parents (Fig. 5).

Another recognized property of cytokine networks is functional redundancy, wherein two independent mediators can fulfill the same role, thereby physiologically compensating for each other’s absence. In both *in vivo* scenarios, IL-4 was peripheral to the main Bayesian network despite tracking IL-13 levels, as established by the cluster analysis. This feature suggests functional redundancy, which is consistent with the fact that IL-4 and IL-13 are known to operate through the same receptor system [9, 14, 65]. It is worth noting that IL-6 and G-CSF were also orphaned in the pup-free dam network. It is unclear whether this reflects true functional redundancy rather than simply the smaller sample size used to construct this particular network. However, even in the lactation network where a larger overall sample size was used, the edges connecting IL-6 and G-CSF to the rest of the network were of low confidence. This highlights the *caveat* that the interpretation of Bayesian networks can benefit from additional *a priori* functional knowledge of the mediators investigated and that larger data sets - unsurprisingly - generate models which more accurately represent mediator inter-relationships, as demonstrated by the use of VBSSM models.

Another interesting phenomenon noted was the response of MIP-1β to changes induced by *in silico* eotaxin manipulation. Intriguingly, perturbing eotaxin to either high or low concentrations both resulted in MIP-1β concentration being higher. Concentration-dependent paradoxical effects are well recognized among cytokines and, in this instance, MIP-1β responses point to a concentration-dependent biphasic response to eotaxin. The implications of this observation are currently unclear but we speculate that this relationship allows scope for homeostatic control, possibly through the existence of physiological feedback loops undetectable using Bayesian methodologies. Alternatively, this may, as outlined for KC, reflect the influence of unknown mediators not measured as part of this investigation.

The broad conservation of cytokine relationships across physiological scenarios points to their integrated regulation and indicates that changes in a small number of driver nodes can potentially affect the concentration of multiple downstream mediators. Given their critical role in orchestrating cytokine networks, identifying driver nodes such as IL-12 (p40), IL-13 and MCP-1 may prove valuable in the exogenous manipulation of inflammatory networks. Major, more predictable network changes could thus be induced through the selective targeting of driver nodes (e.g. by using antibody-based interventions). Moreover, desired cytokine level modulation could be induced without causing major network disruptions in instances where terminal nodes are targeted instead. This would be consistent with current clinical practices such as the use of anti-TNF therapy in the management of a range of autoimmune disorders [66–68]. Interestingly, comparative trials of various agents in rheumatoid arthritis suggest that agents such as these (e.g. etanercept, certolizumab) are superior to those targeting other cytokines such as IL-1 (e.g. anakinra) [69], which reside further upstream in our models. If analogous networks could be constructed using clinical data, there would be scope for developing novel, targeted therapeutic interventions with more predictable immune-related side effects.

This Bayesian network-based analysis has proved valuable in clarifying the complex structure and causal murine systemic cytokine-hormone network relationships. However, these findings must be interpreted in the light of certain *caveats*. Firstly, the present Bayesian networks are necessarily incomplete by virtue of the fact that they do not contain the entire array of possible interacting mediators. Secondly, the interpretation of predicted qualitative changes in network behavior must be considered in a context-specific manner in order to glean physiologically meaningful inferences from the data (e.g. in relation to specific pathophysiologies or in a whole animal instead of cell-specific *in vitro* models which differ in functional receptor expression/cytokine production profiles). Thirdly, they do not represent all possible edges given that the methodology inherently precludes the use of structural loops [25]. This accounts for why the present networks consistently feature TNF-α as the terminal node, despite studies indicating that, for example, TNF-α can induce MIP-1β expression in mice [70]. Fourthly, these networks allow investigators to make valuable predictions about causality, although these may still require empirical verification through specific experiments *in vivo* and/or *in vitro* (e.g. by using specific exogenous cytokines, inhibitors/traps, antibodies, pharmacological agents, hormones, pathogens or physiological insults). Nevertheless, the present findings are, to the best of our knowledge, the earliest available data providing a detailed characterization and assessment of cytokine networks in a whole animal model. The networks generated were statistically robust and independently corroborated many established cytokine interrelationships described in the literature (e.g. between IL-13 and MCP-1, between IL-12 (p40) and IFN-γ) [61, 71].

## Conclusions

The identification of synergy, antagonism, functional redundancy and concentration-dependent biphasic responses within these networks lifts this method of analysis from being purely descriptive to mechanistic. This suggests that Bayesian *in vivo* cytokine networks as shown herein describe real physiological changes in a non-biased fashion, in contrast to modeling endeavors performed on *in vitro* systems (which fall short of presenting a realistic physiological picture) or differential gene expression studies (which do not account for the post-transcriptional regulation of cytokine production) [72]. Furthermore, the identification of conserved regulatory hubs points to the existence of a previously unknown core structure within these cytokine networks whose responses can be predicted with some accuracy. Whilst we believe that the exciting findings of this study are a significant first step towards improving our understanding of complex systemic inflammatory/immune networks, we remain mindful that many of the relationships described herein remain to be individually and more fully validated in the *in vivo* setting. We accept that multiple approaches may be needed in this context in order to build a comprehensive picture of multiple interactions, such as the use of (conditional) knock-out animal models and infusions of cytokine traps, antibodies, cytokines and their soluble/decoy receptors. To this end, our future work will focus on establishing whether the network structures that we have identified herein appear to be conserved across both a range of pathophysiological scenarios (e.g. cancer, autoimmune disorders, cardiovascular disease) as well as across species.

## Methods

### Methodology

Figure 2 demonstrates the workflow used within this study. Initial biological analysis (Experiment 1, see below) investigated the cytokine/hormone profiles in lactating mice, from which Bayesian networks were developed and explored *in silico. In vivo* validation of networks was achieved by investigation of cytokine/hormone networks in non-lactating mice (Experiment 2).

### Animals

#### Experiment 1

Eight to ten-week old virgin CD1 female mice were group housed (10 per cage) with *ad libitum* access to water and Standard Beekay diet (B&K, Grimston, Aldborough, UK). The lighting cycle was 14 h:10 h light:dark, and humidity and temperature were maintained at 55-65 % and 21.5 ± 1 °C. Females were naturally pair-mated to 12–14 week old CD1 stud males of proven fertility following Whitten effect-induced estrus synchronization. Females were caged individually in late pregnancy to litter down and nurse their pups, then sacrificed throughout lactation on days 1 (<24 h of littering), 2, 4, 10, 16, 21 and 24 (*n* = 8, 8, 8, 7, 8, 7 and 7 animals, respectively i.e. 53 data points from which the lactation network was constructed; see later). Weaning occurred on day 21, when the independent pups were removed from their mothers. Samples were collected ±1 h half way through the lighting cycle to minimize the impact of circadian rhythms on any analytes measured. The number of pups per dam was adjusted to 8 by cross-fostering to standardize the suckling stimulus. Negative (baseline) controls were provided by naturally cycling virgin females of the same age and strain (*n* = 7).

#### Experiment 2

An independent data set to test the predictive power (thereby providing biological validation) of the lactation Bayesian network was generated by preventing the establishment of lactation in dams whose entire litters were removed from them at birth (thus maintaining low PRL levels). These females were sacrificed on days 2 and 4 (time-matched) (*n* = 8 in both groups; these 16 data points were used to construct pup-free networks). Seventy-six mice were used in total.

### Ethics statement

The animals used in this study were sacrificed under Schedule 1 of the Animals (Scientific Procedures) Act, 1986 (UK). The use of different animals for each individual time point was required on both ethical and biological grounds given the severe physiological repercussions of collecting blood from lactating dams at such closely spaced time intervals. On one hand, this would have been inappropriate in causing unnecessary ‘pain, distress and lasting harm’ in the eyes of the UK legislative framework. On the other, significant, repeated blood loss would have upregulated both adrenocorticotropic activity and the production of haematopoietic endogenous colony stimulating factors, thereby affecting cytokine networks as a result of sampling rather than true changes in physiology. Moreover, dams subjected to such repeated stress would have been more prone to pup cannibalism, resulting in uneven suckling stimuli across females.

### Sample collection and analysis

For the sake of text readability, cytokine and hormone acronyms are listed in the abbreviations section. Whole blood was collected by cardiac puncture as previously described [73], allowed to clot on ice and serum isolated by centrifugation at 5,000 rpm for 3 min. Serum was stored at −80 °C until analysis. The panel of cytokines chosen was based on the widest murine analytical array (with known immunoendocrine interactions) commercially available at the time of the study. Serum samples were analyzed for IL-1α, IL-1β, IL-2, IL-3, IL-4, IL-5, IL-6, IL-9, IL-10, IL-12 (p40), IL-12 (p70), IL-13, IL-17, eotaxin, G-CSF, GM-CSF, IFN-γ, KC, MCP-1, MIP-1α, MIP-1β, RANTES and TNF-α by multiplex immunoassay (Bio-Rad Laboratories, Hemel Hempstead, Hertfordshire, UK) on a Luminex-100 cytometer (Luminex Corporation, Austin, Texas), equipped with StarStation software (Applied Cytometry Systems, Dinnington, UK), as previously described [9]. Samples were analyzed on one plate to avoid any potential batch or inter-plate variation. No missing values were identified, and samples falling below the level of detection of the assay were allocated a concentration of 0 pg/ml in relation to the blank in order to avoid skewing the data sets to an unrepresentative higher concentration. Hormones relevant to lactation with putative immunomodulatory effects were also selected for analysis: PRL concentrations were determined by homologous specific radioimmunoassay [74], while E_2_ and P_4_ were assayed by enzyme-linked immunosorbent assay according to the manufacturer’s instructions (Alpha Diagnostic, San Antonio, Texas).

### Data presentation and analysis

Data were expressed as pg/ml (cytokines, E_2_) or ng/ml (PRL, P_4_) ± SEM. All data distributions were assessed for normality by Anderson-Darling tests. Basic analytical approaches were performed to highlight time course-related changes in analyte profiles and to better appreciate the data before applying machine learning approaches. These were based on subsequent Kruskall-Wallis/analysis of variance with *post hoc* Mann–Whitney-*U*/Fisher’s LSD tests, as appropriate. Pup removal data were similarly compared using t-tests or Mann–Whitney-*U* tests. Corrections for multiple comparisons were applied using the Benjamini and Hochberg False Discovery Rate method [75]. Statistical analyses were performed using Minitab (Version 16) and ‘R’.

In order to identify significant changes in mean concentration of each cytokine over time throughout lactation, *z*-scores were computed for each of these, assuming a null hypothesis that the variable was constant at the weighted mean value, where the weight was (standard error)^−2^ i.e. the inverse square of the standard error (estimated for each cytokine/hormone at each time point using the available multiple measurements). Resultant *z*-score *P* values (0.05 threshold) were corrected using the False Discovery Rate. Time-series were also analyzed using Bayesian Hierarchical Clustering (BHC) in order to define the number of data clusters in a principled manner, wherein the BHC algorithm identified distinct groupings solely on the basis of input data [76]. They were modeled as being drawn from one of a number of underlying curves and assigned to a specific cluster on strictly probabilistic grounds. Since the time-series were normalized to have zero mean and unit variance, the clustering analysis was sensitive only to their shape. A flexible, non-parametric regression Gaussian Process Model (herein inferring a nonparametric latent function over time) was fitted to the mean at each time point in order to model the trend underlying mean value changes over time for each mediator. Correlations between cytokine, steroid hormone and PRL profiles were determined using Pearson’s product–moment correlations as a basis for the heat map.

### Bayesian network construction

In Bayesian network formalism, a network of interacting variables is represented as a graph in which the variables are nodes and their interactions are directed edges [18]. The edge between two nodes, P_1_ and P_2_, is associated with a conditional probability table containing probability of the state of P_2_ given the state of P_1_. The approach only allows dependencies between a node and its immediate parents. Formally, a Bayesian network is defined to be a pair (G, Θ_G_) where G is a directed acyclic graph whose vertices are random variables P_i_ and Θ_G_ is the conditional distribution for each variable given its parents: P_b_(P_i_ | P_a_(P_i_)), where P_a_(P_i_) denotes the set of all parents of P_i_ in the graph. Conditional independence statements, encoded by the network structure, define the conditional probability distribution.

In order to establish a prior network containing proteins from the present analytical target set, a seed network learned from the biomedical literature, protein-protein interaction databases, or any combination thereof, was used. The information incorporated in the seed network was solely restricted to well-established mouse-based interactions, given that many cytokines exhibit species-specific differences in function owing to evolutionary adaptation of what is effectively a plastic system with inbuilt functional redundancy. MetaCore Inc. (GeneGo, a Thomson Reuters business, http://thomsonreuters.com/en/products-services/pharma-life-sciences/pharmaceuticalresearch/metacore.html) was principally used to generate the prior network along with thorough hand curation. The resultant seed network was then combined with the results obtained from ‘Predictionet’ (http://www.bioconductor.org/packages/devel/bioc/html/predictionet.html), a text mining web application which retrieves gene interactions reported in the literature by focusing on a core set of targets and the context within which the information pertaining to them is retrieved/based [77]. Any conflicting edges in the prior network causing feedback cycles were removed since Bayesian networks inherently preclude the existence of structural loops: no node/child can be either its own ancestor or descendant (analogously, the prior of the network structure could not contain such loops either as it was incorporated as a multiplicative factor in the scoring metric). The network structure close to the prior network has higher probability: the parametric formula for this prior structure-related factor in the scoring metric is given in Heckerman et al. (1995) [24]. Prior to performing the Bayesian network analysis, *z*-score normalizations were applied to the raw data in Matlab. A machine learning algorithm (implemented in the WEKA-based open-source package MeV [78]; http://www.cs.waikato.ac.nz/ml/weka/) was used to refine the seed network in conjunction with the experimental data derived from postpartum mice in order to predict a high-confidence network [21, 79]. We found that the literature mining and the protein:protein database (PPI) toolbox embedded within the MeV package proved largely ineffective for establishing a seed network.

Cytokine and hormone profiles were further discretized into categorical data for the BN analysis following *z*-score application in order to reduce computational expense, making these relative over time courses and thus a prerequisite for assessing network behavior, which is based on relative rather than absolute concentrations. These were assigned to three mutually exclusive equal width relative concentration bins i.e. rescaled/normalized data spreading between 0 and 1 were categorized into low, intermediate/neutral and high bands of different sizes but with the same frequency such that the number of samples allocated to each category was the same. Thus, in the learned networks, each protein had an underlying conditional probability table where the color of each node was determined by its own allied underlying histogram. The snapshot of a network thus represents *relative* (rather than absolute) concentrations of each protein in a given physiological setting (i.e. lactation or pup-free) following the independent categorization described above for each dataset.

Time-dependent autocorrelations may have been present across different temporal measurements in each series (since the data were drawn from a time course) such that there was a violation of the static BN learning assumption that training samples are independent. However, an assumption of independence between time points is less problematic in defining the 3 bin-based concentration states than the first-order Markov assumption in a typical dynamic state space model on continuous data. Moreover, the Spearman correlation coefficient between two nodes is significantly reduced when the system is randomly sampled over time with fewer time points. Hence, a static Bayesian network structure learning is expected to provide a more appropriate model for sparsely sampled data, as is often the case in a biological context. On the other hand, static modelling for a time series data is more meaningful under the assumption that the effects exist long enough to be visible across multiple time steps, such that causal relationships are sustained rather than one-off events. The Spearman correlation coefficients between two causally connected nodes remained high in both short and long sampling schemes. As regards data discretization, even in the presence of a periodic cycle, it would have been valid to apply this to concentration differences for the purposes of training a relationship model involving multiple variables. These data were then used to learn the Bayesian network. Both the network topology and edge-specific conditional probabilities were learned from the data, starting from the initial seed network. Only nodes with three parents were selected, as per convention in the field [21, 80]. Searching for a best possible network for a given set of moderate numbers of proteins or genes is computationally expensive. Among the various simplifying assumptions to tackle this problem, one is to reduce the number of parameters associated with the conditional probability tables for each non-root node by restricting the maximum number of parent nodes for a given child to be three - a reasonable assumption in a biological context. However, in the present study, the very high bootstrapping stringency resulted in more than three parents for some nodes in the final network. In this regard, standard non-parametric bootstrapping was applied (100 operations) to address potential over-fitting in the Bayesian analysis, wherein multiple data sets were created by re-sampling with replacement to estimate the confidence in the various network features, such as edges between the nodes learned [81]. Several metrics and search algorithms were employed in combination in order to optimize the sensitivity of the results to the learning procedure scoring metric [82]; the Tabu Search algorithm was used to optimize the Bayes (BDe) score as the selected scoring metric.

Overall, 53 data points were used in total but none of the 81 cells filled by the prior probabilities were zero: this is inherent to the Bayesian metric with Dirichlet prior distribution (the Bayes and the special case BDe metric) structure. The scoring metric contains both the coefficients denoting the number of records in the dataset and the choices of priors on the counts coming from the Dirichlet distribution in order to fill all 81 cells. The assumptions behind the Bayesian measure of the goodness of a belief-network structure lead to strong constraints on the exponents so that all of them can be constructed [24]. For a brief description of the Bayes and its special case the BDe metric, and the Tabu search algorithm used herein, please refer to the Weka manual: http://weka.sourceforge.net/manuals/weka.bn.pdf.

Bayesian networks represent a ‘snapshot’ at any given time; however, the networks generated herein reflected sustained effects or causal events seen across multiple time points. For Bayesian network generation, the interaction matrix between each node for each time point was learned and then used to initialize the inference process for the next time point. Therefore, the final Bayesian network visualized from the final time point reflected the dynamic process encoded by the previous time step. Overall, therefore, the structure of seeded networks was learned both from the data and prior knowledge network, which biased the search to a subspace. This way of optimization is an alternative to heuristic methods which avoids the likelihood of hitting local minima.

The robustness of the present approach was ensured by the bootstrapping experiment outlined above which provided good feature confidence measures and returned few low confidence edges, even after increasing the stringency of the bootstrap confidence to 0.9 (i.e. features occurring in ≥90 % of iterations). The network directed acyclic graph was then visualized using Cytoscape (http://www.cytoscape.org) [83].

#### VBSSM model generation

In order to further validate the robustness of this model, a VBSSM was built in Matlab [77]. VBSSM implements an analytical approximation scheme to Bayesian state-space models and, unlike other related methods, does not take prior information (i.e. the seed network) into account for network reconstruction [84], such that networks were solely constructed from the experimental data. It should be noted that although time t + 1 involved 7–8 replicates independent from those of time t, these experiments used mice of the same strain/age with even litter sizes whose lactation started at approximately the same time under identical conditions. In this way, the sets of replicates across time points were assumed to be dynamically related so as to make VBSSMs meaningful for the purposes of internal, independent validation of the models learned using a seeded Bayesian network method.

VBSSM is a dynamic Bayesian network inference algorithm that uses linear Gaussian state-space models to help reverse-engineer interactions between proteins or transcriptional networks from time series data, thereby explicitly modelling the progression of a system over a multi-step time course. In state-space Bayesian models, the observed measurements depend on hidden states, which are assumed to evolve according to first order Markov chain. A variational approach to the above models presents a novel way to learn the structure and the optimal dimensions of the state space and, as such, the VBSSM algorithm provides distributions over the model parameters leading to an inference of its underlying structure. The majority of algorithms, including VBSSM, only allow edges across time steps and transition models that are stationary (linear time invariant) and first order Markovian. It is relatively efficient in terms of the size of the network search space and does not require sub computations, such as checking cycles within the network. However, the assumption of a stationary, first order Markov model is not appropriate for sparsely sampled datasets with a small number of time points each featuring a limited number of biological replicates as in the present data sets. For example, if node A inhibits B, the corresponding high negative Spearman correlation coefficient is significantly reduced when the system is randomly sampled over time with fewer time points. Hence, the traditional static Bayesian network structure learning is expected to provide a more appropriate model for sparsely sampled data (as is frequently the case in a biological context), which accounts for the use of static networks described above. Nevertheless, static modelling for a time series data is more meaningful under assumption that the effects exist long enough to be visible across multiple time steps and causal events are sustained causes, not one-shot triggers. The Spearman correlation coefficients between two causally connected nodes remain high in both short and long sampling schemes. Since the data are drawn from a time course, it invalidates the static Bayesian network learning assumption that training samples are independent. However, due to the nature of the sampling process, it is believed that a violation of this assumption is less problematic than the first-order Markov assumption.

In the present study, the VBSSM was constructed from 6 randomly chosen replicates from each time point (as there were 7–8 total replicates for each time point). The interaction matrix between nodes for each time step was learned and used to initialize the inference process for the next time step. In order to get the final static snapshot of the Bayesian network, the network was taken from the final time step, which takes into account the history of the dynamic process as encoded in the networks from previous time steps; recall, as outlined above, that VBSSMs are linearly time invariant.

The VBSSM algorithm allowed for the selection of a high significance level network interactions (measured in terms of *z*-score). In this regard, the *z*-score was used to calculate the normal cumulative distribution *p*, *viz*. that the probability that an observation from a normal distribution with mean zero and variance one will be less than *z*, giving a significance level of 1-*p*. Accordingly, a larger *z*-score means a lower significance level and a more stringent requirement level for the edges and nodes in a derived network [77]. The networks obtained herein had a *z*-score of 2.33, which corresponded to a significance level of 0.01 and cumulative probability of 0.99.

### Bayesian network perturbation and *in vivo* validation

Based on the results obtained in the first network, the systematic *in silico* perturbation of eotaxin, IL-3, IL-12 (p40), IL-13, MCP-1 and PRL nodes was performed by altering their conditional probability table values (thereby removing the uncertainties of concentration status) in order to determine their relative contribution towards the cytokine network structure [63]. PRL (the key expected change between both experiments which would offer biological validation of *in silico* predictions) was perturbed first by allocating it to a low concentration bin (probability of 1) and the effects of this operation recorded as conditional probability tables which represented shifts in concentration (i.e. bin conditional probability) status of downstream nodes. This enabled us to determine its *positive* effects on immediate downstream hub nodes. Thereafter, as main first network downstream branch hubs from PRL, IL-13 and MCP-1 were perturbed (given a high concentration status), both individually and in combination. As a final step, IL-3 and IL-12 (p40) were perturbed alone and in combination with IL-13, MCP-1 and PRL in various combinations in order to determine their relative interactions and contributions to the network structure. The changes in conditional probabilities for downstream nodes elicited by these *in silico* perturbations were then compared to those obtained *in vivo* following pup-removal (which naturally abrogated lactation and thus prevented a rise in PRL).

### Network structural comparisons

*In silico* network perturbations preserved network structure as they only propagated the disturbance along a fixed network topology. By contrast, *in vivo* perturbation (i.e. pup removal) resulted in network topological changes, as anticipated given that the second network was constructed from an independent data set. The R package *Catnet* (http://cran.r-project.org/web/packages/catnet/index.html), a categorical Bayesian network inference framework was used to systematically assess the structural similarities between the lactation and pup-free networks by computing the F-score, which represents the harmonic average of specificity and sensitivity, and accounts for inter-structural edge appearances/disappearances. It is expressed in terms of the number of true positive (TP), true negative (TN), false positive (FP) and false negative (FN) edges, and represents a statistical score of similarity between any two networks in terms of edge connection features. In this instance, TP, TN, FP and FN refer to the directed edges of the *in vivo* perturbed network with respect to the lactation network (taken as the standard of truth). The following formula was used:

$$ \mathrm{F}\hbox{-} \mathrm{score} = 2\times \mathrm{specificity}\times \mathrm{sensitivity}/\left(\mathrm{specificity} + \mathrm{sensitivity}\right) $$

Where: specificity = TN/(TN + FP)

sensitivity = TP/(TP + FN)

As an additional measure of network similarity, the comparative complexity of these networks was also assessed as a representative measure given that this feature depends on both graphical structure and node categorization, and thus hinges on the number of parameters needed to define network probability distributions. The present Bayesian networks are categorical (i.e. discrete), with each node being assigned a value in a fixed set of categories. Total network complexity was thus measured as the sum of all of its node complexities, which were individually determined by the number of their respective parents and categories. A node with k parents with respective number of categories c_1_, c_2_, .., c_k_ has complexity c_1_ x c_2_ x … x c_k_. Although in the original analysis the networks constructed were category 3 with 3 bins for evaluating each node’s conditional probability, category 2 was chosen herein during the simulation process for simplicity. This change in category did not affect any of results obtained because only structural features, in particular topological similarity between the different networks, were being investigated.

It is worth noting that the discretized concentration distributions and binning were independent for the lactation and pup-free data sets. This was deliberate given that, firstly, relative concentrations define a system’s behavior rather than absolute values [85], making it more meaningful to compare relative nodal statuses across models, especially if covering different time courses (and thus tailored to different biological endpoints as herein). Secondly, for unbiased model validation, it is crucial that the validation data set should not inform model development based on the training data set (a paradigm which would be have been violated had the categories been defined on the basis of pooled data).

## Additional files

Additional file 1:
**Changes in cytokine and hormone profiles throughout lactation; Cytokine, E**_**2**_ **(pg/ml), PRL and P**_**4**_ **(ng/ml) concentrations throughout lactation (mean ± SEM).** Groups that do not share a common superscript letter are significantly different from each other (*P <* 0.05). NC - naturally cycling. (TIF 644 kb) Additional file 2:
**Prior network utilised to develop the Bayesian network.** The prior network was learned from the literature relating to murine cytokine interactions only. Nodes are displayed within circles, with edges representing known interactions. Due to the nature of the discovery of the prior network, edges do not represent directionality. (TIF 1064 kb) Additional file 3:
**Circular variational Bayesian state-space model (VBSSM) of cytokine interactions during physiological lactation; Although it was created entirely from the experimental data with no inferences drawn from prior knowledge, much of the core nodal structure present in the seeded network also emerges in this acyclic graph (A).** Circular VBSSM of cytokine interactions in the pup-free group. The nodes p40 and p70 refer to the IL-12 (p40) subunit and (p70) heterodimer, respectively (B). (TIF 914 kb) Additional file 4:
**Bayesian lactation network perturbation by deterministically decreasing PRL concentration; Includes conditional probability indicators for each component node (color coding is as described for Fig.** **3** **in the main text).** Bars beside each node represent conditional probabilities (low to high, from right to left). (TIF 3620 kb) Additional file 5:
**Bayesian lactation network perturbation by deterministically increasing IL-13 concentration (A), MCP-1 concentration (B) and in combination (C).** (TIF 3966 kb) Additional file 6:
**Bayesian lactation network perturbation by PRL branch (combined PRL/IL-13/MCP-1) perturbation.** (TIF 6170 kb) Additional file 7:
**Bayesian lactation network perturbation by deterministically increasing IL-12 (p40) concentration alone (A) or in conjunction with increasing MCP-1 (B).** (TIF 2996 kb) Additional file 8:
**Bayesian lactation network perturbation by deterministically increasing IL-12 (p40) concentration in the presence of decreased (A) or increased (B) eotaxin.** (TIF 3060 kb) Additional file 9:
**Changes in conditional probability associated with perturbation of eotaxin and** IL-12 **(p40) as parent nodes relative to those induced by perturbing** **MCP-1** **and** **IL-13** **as hubs.** Subscripts indicate the direction of the perturbation (−low, +high). (DOCX 14 kb) Additional file 10:
**Conditional probabilities associated with IL-3 perturbation.** Subscripts indicate the direction of the perturbation (L-low, M-Medium, H-high). (DOCX 14 kb) Additional file 11:
**Bayesian lactation network perturbation by deterministically increasing (A) and decreasing (B) eotaxin concentration.** (DOCX 14 kb)

## Abbreviations

BHC: Bayesian hierarchical clustering
E_2_: estrogen
G-CSF: granulocyte-colony stimulating factor
GM-CSF: granulocyte macrophage-colony stimulating factor
IL: interleukin
IFN: interferon
KC: keratinocyte chemoattractant
MCP: monocyte chemoattractant protein
MIP: macrophage inflammatory protein
P_4_: progesterone
PRL: prolactin
RANTES: regulated upon activation, normal T cell expressed and secreted
SE: standard error
SEM: standard error of the mean
TNF: tumor necrosis factor
VBSSM: Variational Bayesian state space model
