# Supplementary figures and images for: Bayesian modeling suggests that IL-12 (p40), IL-13 and MCP-1 drive murine cytokine networks in vivo
Source: BMC Syst Biol. 2015 Nov 9;9:76. doi: 10.1186/s12918-015-0226-3 (PMC4640223; doi:10.1186/s12918-015-0226-3)

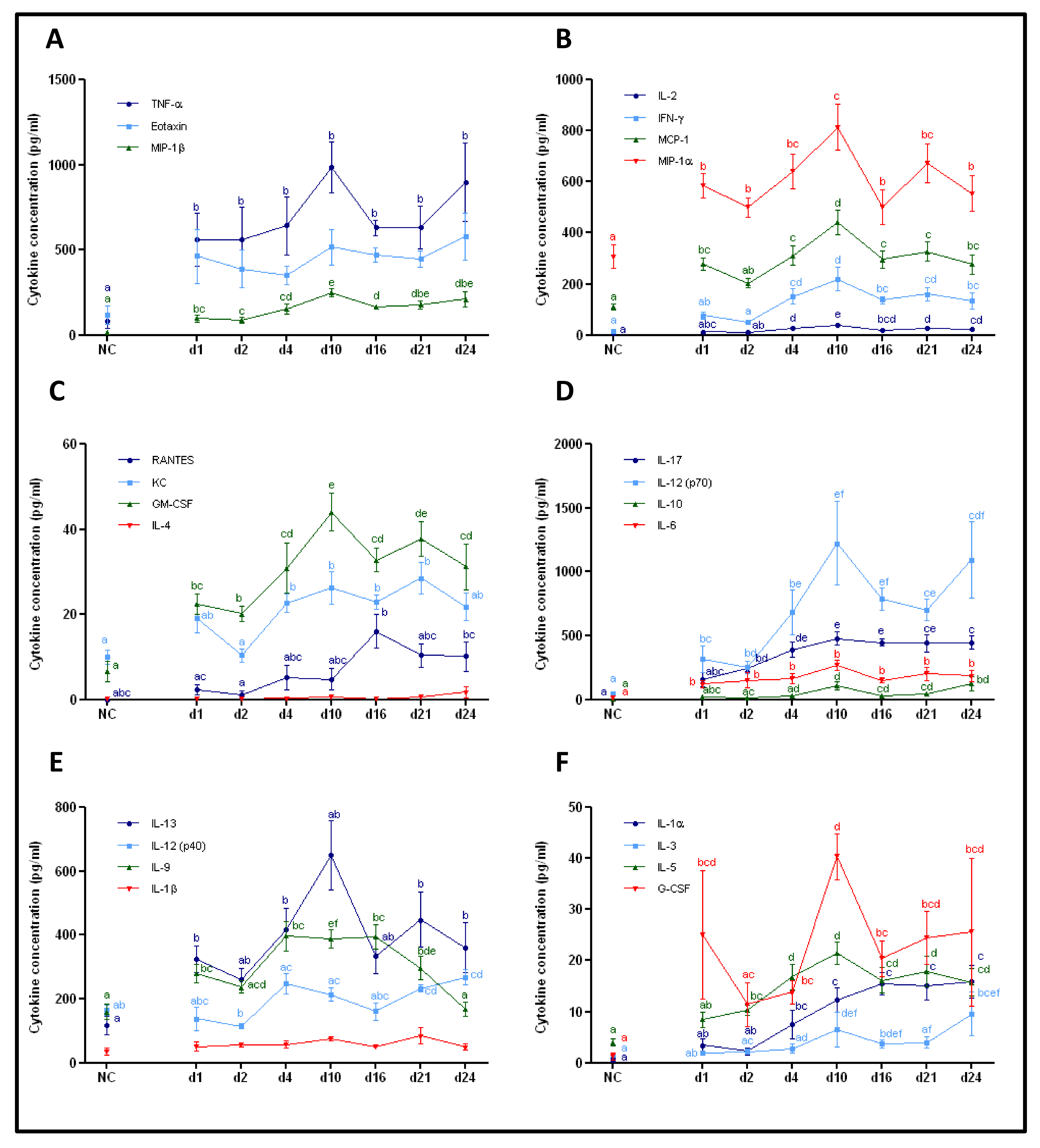

Supplement: Additional file 1: — Changes in cytokine and hormone profiles throughout lactation; Cytokine, E 2 (pg/ml), PRL and P 4 (ng/ml) concentrations throughout lactation (mean ± SEM). Groups that do not share a common superscript letter are significantly different from each other (P < 0.05). NC - naturally cycling. (TIF 644 kb) [file 12918_2015_226_MOESM1_ESM.tif]

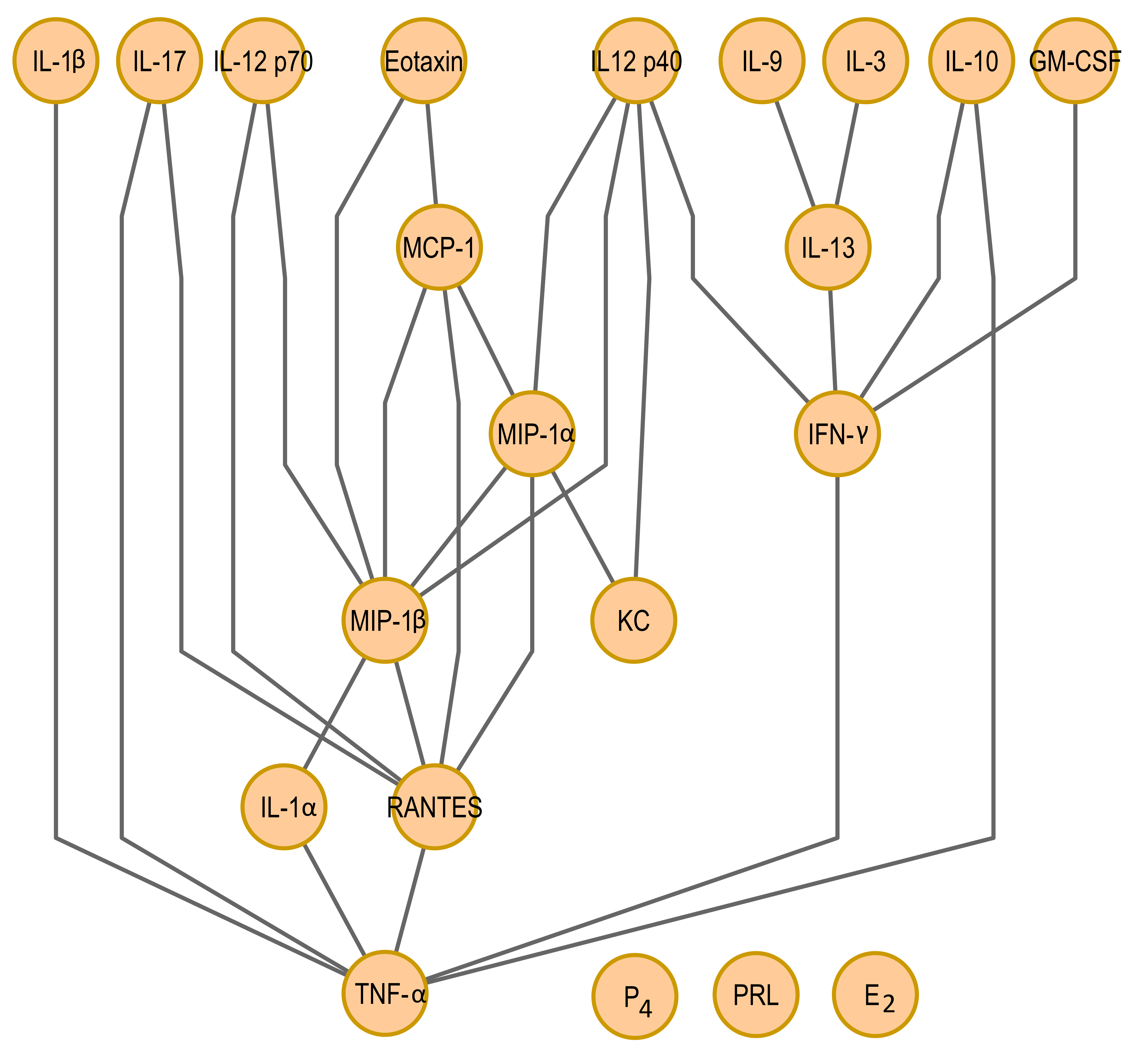

Supplement: Additional file 2: — Prior network utilised to develop the Bayesian network. The prior network was learned from the literature relating to murine cytokine interactions only. Nodes are displayed within circles, with edges representing known interactions. Due to the nature of the discovery of the prior network, edges do not represent directionality. (TIF 1064 kb) [file 12918_2015_226_MOESM2_ESM.tif]

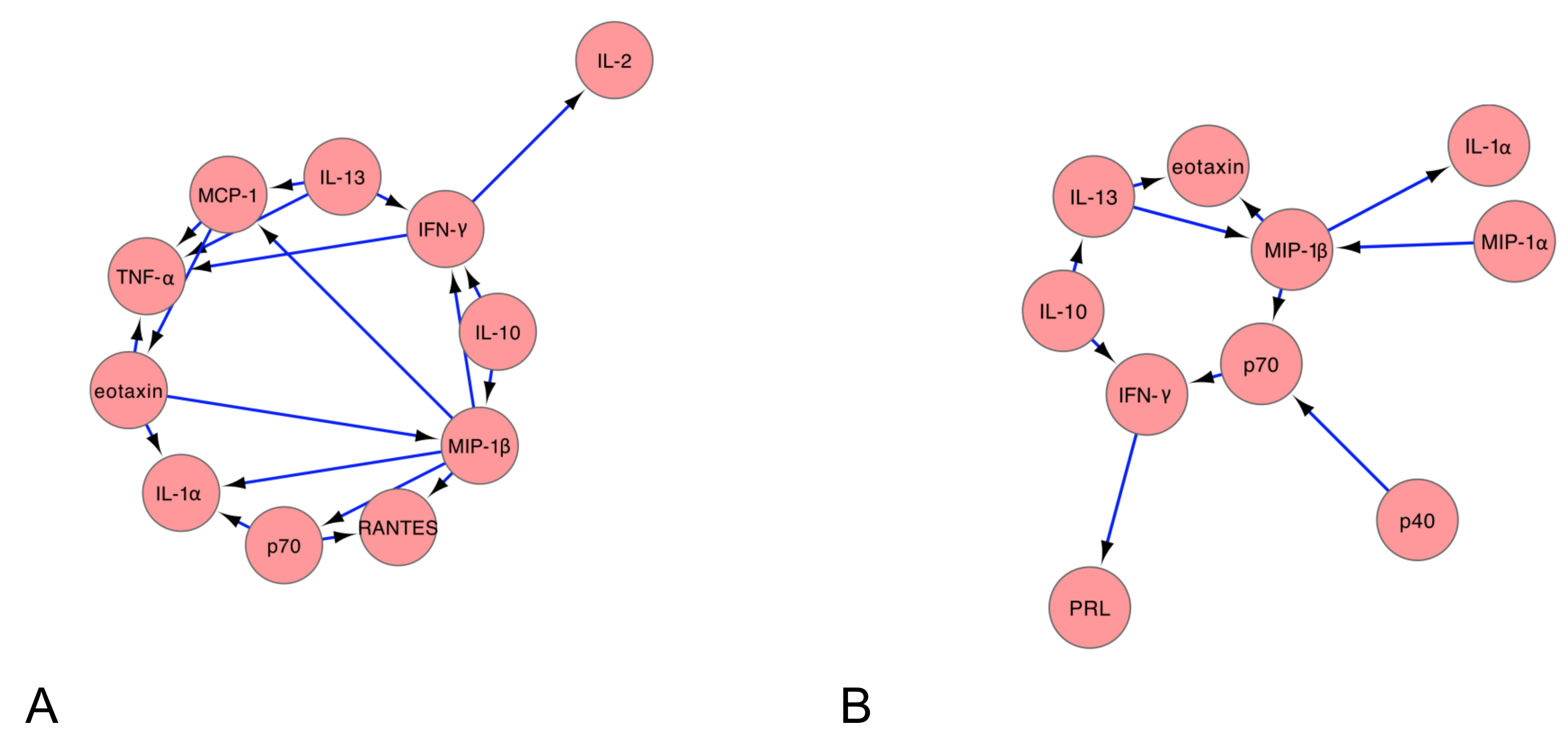

Supplement: Additional file 3: — Circular variational Bayesian state-space model (VBSSM) of cytokine interactions during physiological lactation; Although it was created entirely from the experimental data with no inferences drawn from prior knowledge, much of the core nodal structure present in the seeded network also emerges in this acyclic graph (A). Circular VBSSM of cytokine interactions in the pup-free group. The nodes p40 and p70 refer to the IL-12 (p40) subunit and (p70) heterodimer, respectively (B). (TIF 914 kb) [file 12918_2015_226_MOESM3_ESM.tif]

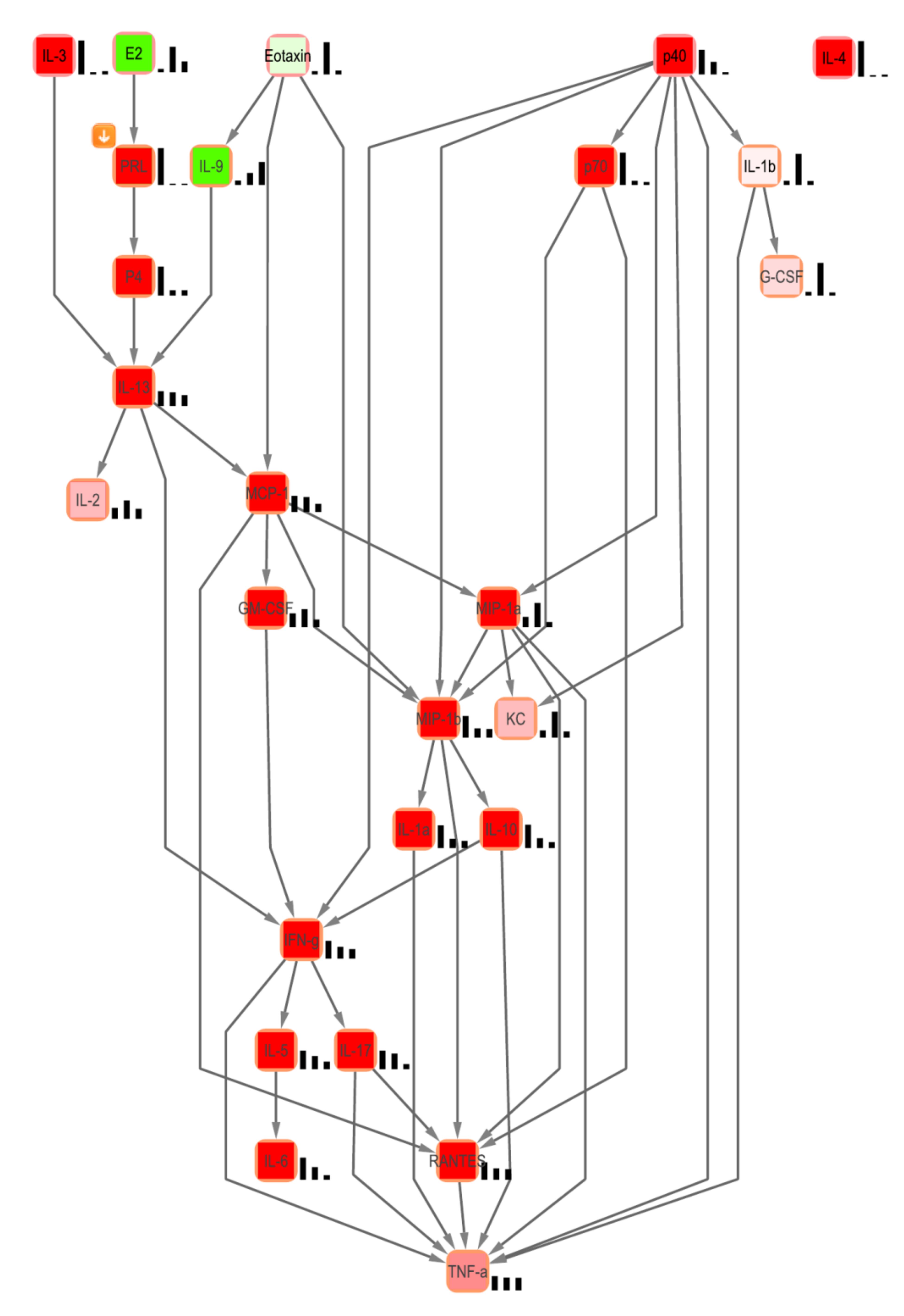

Supplement: Additional file 4: — Bayesian lactation network perturbation by deterministically decreasing PRL concentration; Includes conditional probability indicators for each component node (color coding is as described for Fig. 3 in the main text). Bars beside each node represent conditional probabilities (low to high, from right to left). (TIF 3620 kb) [file 12918_2015_226_MOESM4_ESM.tif]

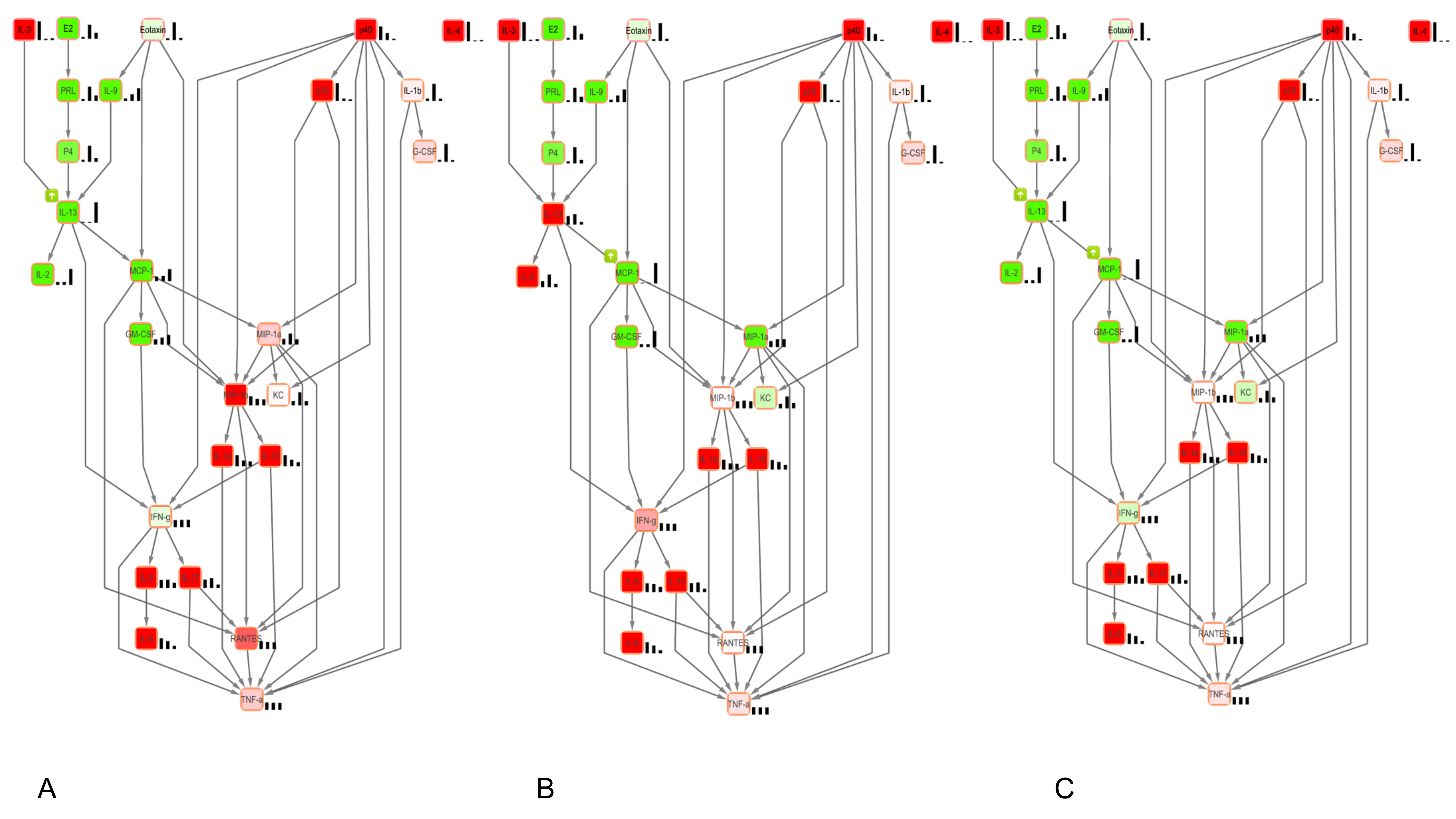

Supplement: Additional file 5: — Bayesian lactation network perturbation by deterministically increasing IL-13 concentration (A), MCP-1 concentration (B) and in combination (C). (TIF 3966 kb) [file 12918_2015_226_MOESM5_ESM.tif]

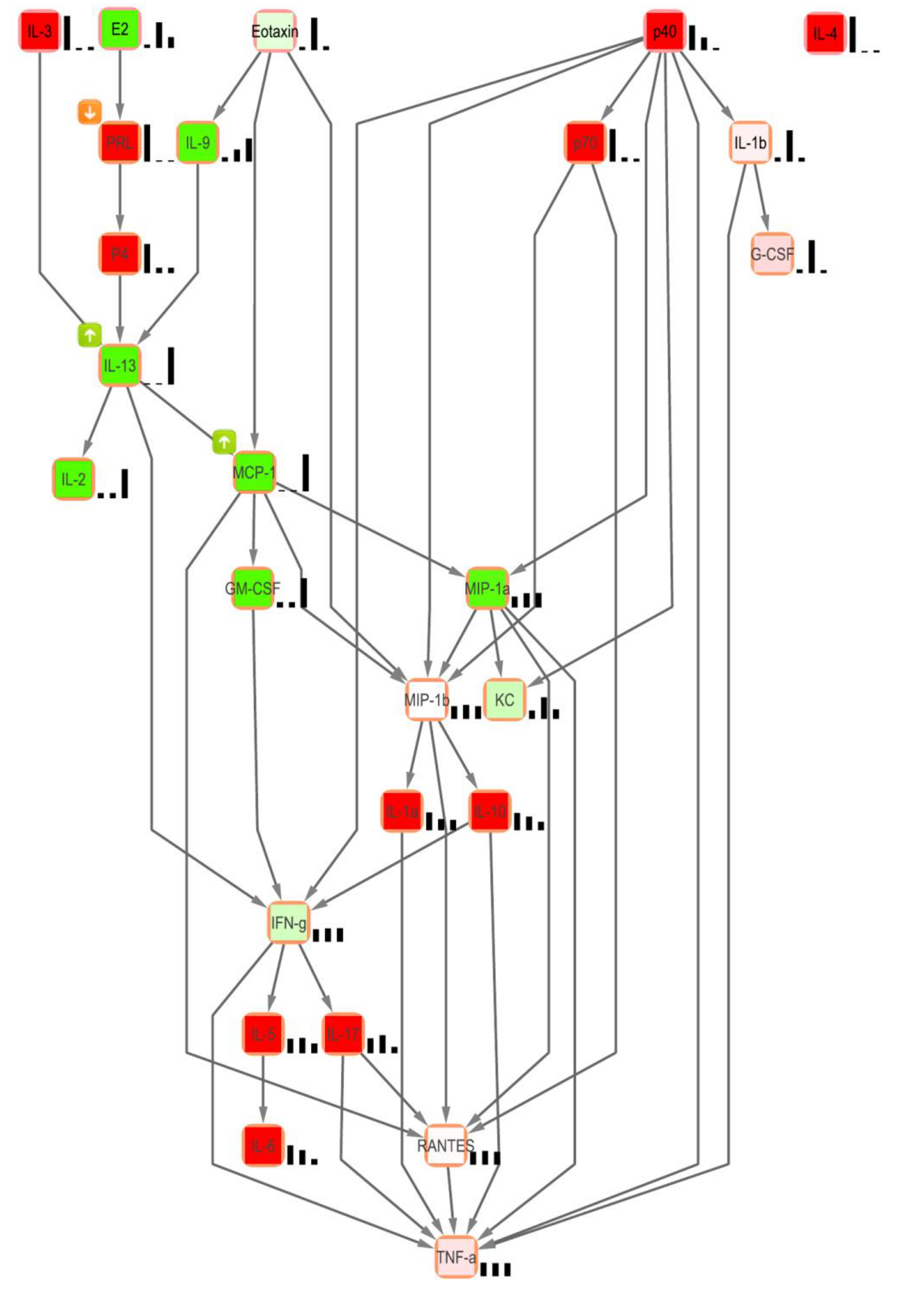

Supplement: Additional file 6: — Bayesian lactation network perturbation by PRL branch (combined PRL/IL-13/MCP-1) perturbation. (TIF 6170 kb) [file 12918_2015_226_MOESM6_ESM.tif]

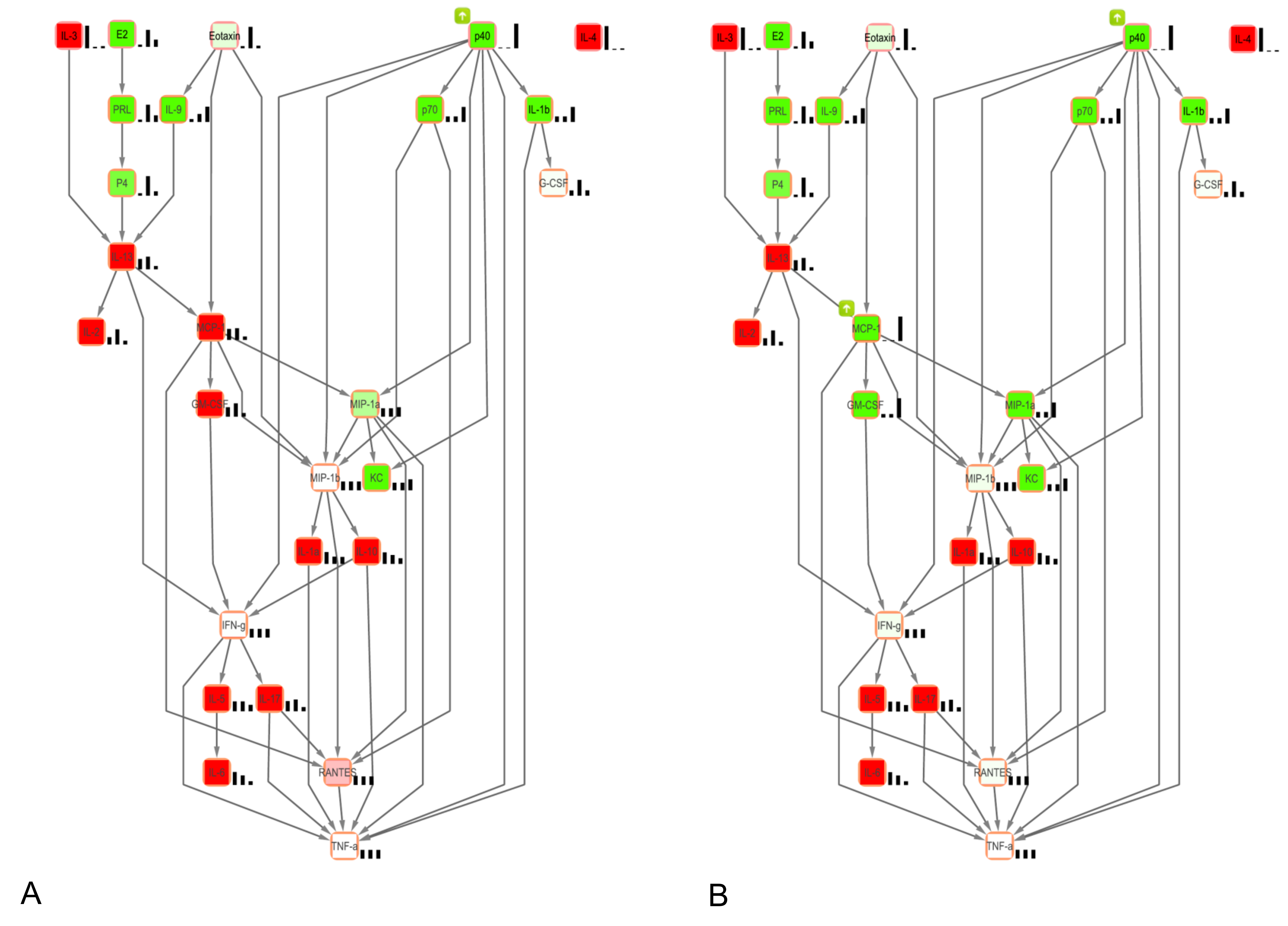

Supplement: Additional file 7: — Bayesian lactation network perturbation by deterministically increasing IL-12 (p40) concentration alone (A) or in conjunction with increasing MCP-1 (B). (TIF 2996 kb) [file 12918_2015_226_MOESM7_ESM.tif]

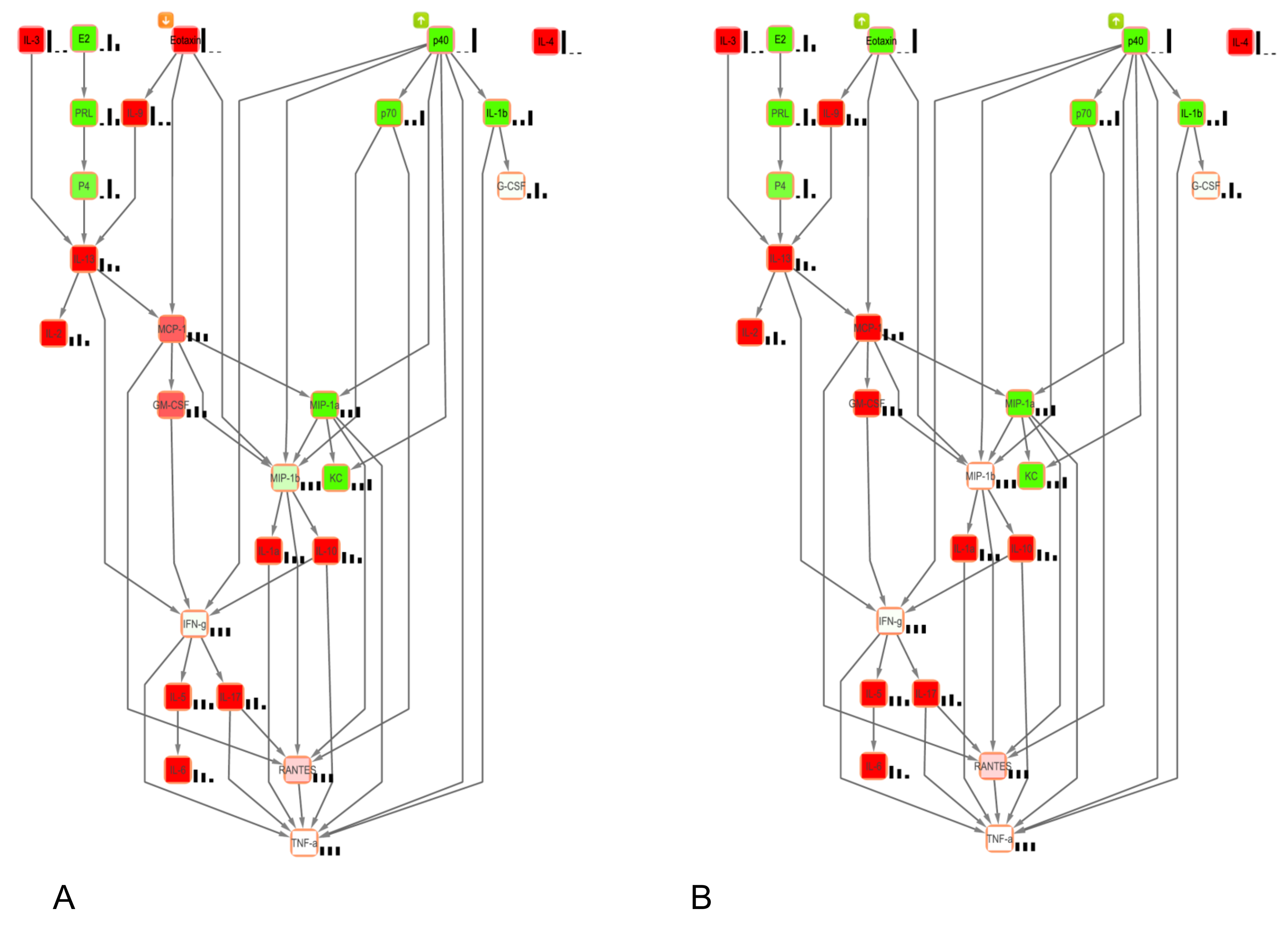

Supplement: Additional file 8: — Bayesian lactation network perturbation by deterministically increasing IL-12 (p40) concentration in the presence of decreased (A) or increased (B) eotaxin. (TIF 3060 kb) [file 12918_2015_226_MOESM8_ESM.tif]
