# Supplementary material for: Bayesian modeling suggests that IL-12 (p40), IL-13 and MCP-1 drive murine cytokine networks in vivo
Source: BMC Syst Biol. 2015 Nov 9;9:76. doi: 10.1186/s12918-015-0226-3 (PMC4640223; doi:10.1186/s12918-015-0226-3)
Supplement: Additional file 9: — Changes in conditional probability associated with perturbation of eotaxin and IL-12 (p40) as parent nodes relative to those induced by perturbing MCP-1 and IL-13 as hubs. Subscripts indicate the direction of the perturbation (−low, +high). (DOCX 14 kb) [file 12918_2015_226_MOESM9_ESM.docx]

**Additional file 9**

| **Cytokines** | **Low** | **Medium** | **High** |
| --- | --- | --- | --- |
| **MIP-1α** | 0.235 | 0.687 | 0.078 |
| **MIP-1α_+IL-12 (p40)_** | 0.319 | 0.319 | 0.363 |
| **MIP-1α_+MCP-1_** | 0.270 | 0.360 | 0.370 |
| **MIP-1α_+IL-12 (p40)+MCP-1_** | 0.200 | 0.200 | 0.600 |
| **MIP-1β** | 0.607 | 0.208 | 0.186 |
| **MIP-1β_+IL-12 (p40)_** | 0.333 | 0.333 | 0.334 |
| **MIP-1β_+Eotaxin_** | 0.323 | 0.368 | 0.309 |
| **MIP-1β_-Eotaxin_** | 0.339 | 0.332 | 0.329 |
| **MIP-1β_+IL-12 (p40)+Eotaxin_** | 0.333 | 0.333 | 0.333 |
| **MIP-1β_+IL-12 (p40)-Eotaxin_** | 0.324 | 0.324 | 0.352 |
| **MIP-1β_+MCP-1_** | 0.321 | 0.357 | 0.321 |
| **MIP-1β_+IL-12 (p40)+MCP-1_** | 0.331 | 0.331 | 0.339 |
| **IFN-γ** | 0.504 | 0.299 | 0.197 |
| **IFN-γ_+IL-13_** | 0.325 | 0.337 | 0.338 |
| **IFN-γ_+IL-12 (p40)_** | 0.333 | 0.333 | 0.334 |
| **IFN-γ_+IL-12(p40)+IL-13_** | 0.321 | 0.321 | 0.355 |
