# Supplementary material for: Bayesian modeling suggests that IL-12 (p40), IL-13 and MCP-1 drive murine cytokine networks in vivo
Source: BMC Syst Biol. 2015 Nov 9;9:76. doi: 10.1186/s12918-015-0226-3 (PMC4640223; doi:10.1186/s12918-015-0226-3)
Supplement: Additional file 10: — Conditional probabilities associated with IL-3 perturbation. Subscripts indicate the direction of the perturbation (L-low, M-Medium, H-high). (DOCX 14 kb) [file 12918_2015_226_MOESM10_ESM.docx]

**Additional file 10**

|  | **Low** | **Medium** | **High** |
| --- | --- | --- | --- |
| **IL-3** | 0.919 | 0.024 | 0.057 |
| **IL-13** | 0.400 | 0.499 | 0.101 |
| **IL-13_IL-3­_L_** | 0.410 | 0.507 | 0.083 |
| **IL-13_IL-3­_M_** | 0.333 | 0.333 | 0.335 |
| **IL-13_IL-3_H_** | 0.273 | 0.446 | 0.281 |
| **IL-2** | 0.274 | 0.602 | 0.124 |
| **IL-2_IL-3_H_** | 0.278 | 0.610 | 0.111 |
| **IL-2_IL-3_M_** | 0.252 | 0.456 | 0.292 |
| **IL-2_IL-3_H_** | 0.215 | 0.526 | 0.258 |
| **MCP-1** | 0.431 | 0.458 | 0.111 |
| **MCP-1_IL-3_L_** | 0.437 | 0.462 | 0.102 |
| **MCP-1_IL-3_M_** | 0.391 | 0.380 | 0.229 |
| **MCP-1_IL-3_H_** | 0.359 | 0.436 | 0.205 |
